# Mutations of Omicron Variant at the Interface of the Receptor Domain Motif and Human Angiotensin-Converting Enzyme-2

**DOI:** 10.3390/ijms23052870

**Published:** 2022-03-06

**Authors:** Puja Adhikari, Bahaa Jawad, Rudolf Podgornik, Wai-Yim Ching

**Affiliations:** 1 Department of Physics and Astronomy, University of Missouri-Kansas City, Kansas City, MO 64110, USA; paz67@umkc.edu (P.A.); bajrmd@umkc.edu (B.J.); 2 Department of Applied Sciences, University of Technology, Baghdad 10066, Iraq; 3 School of Physical Sciences and Kavli Institute of Theoretical Science, University of Chinese Academy of Sciences, Beijing 100049, China; rudipod@gmail.com; 4 CAS Key Laboratory of Soft Matter Physics, Institute of Physics, Chinese Academy of Sciences, Beijing 100090, China; 5 Wenzhou Institute of the University of Chinese Academy of Sciences, Wenzhou 325000, China

**Keywords:** SARS-CoV-2, spike protein, Omicron variant, AABP unit, complexity in shape and volume, partial charge, virial transmission

## Abstract

The most recent Omicron variant of SARS-CoV-2 has caused global concern and anxiety. The only thing certain about this strain, with a large number of mutations in the spike protein, is that it spreads quickly, seems to evade immune defense, and mitigates the benefits of existing vaccines. Based on the ultra-large-scale ab initio computational modeling of the receptor binding motif (RBM) and the human angiotensin-converting enzyme-2 (ACE2) interface, we provide the details of the effect of Omicron mutations at the fundamental atomic scale level. In-depth analysis anchored in the novel concept of amino acid-amino acid bond pair units (AABPU) indicates that mutations in the Omicron variant are connected with (i) significant changes in the shape and structure of AABPU components, together with (ii) significant increase in the positive partial charge, which facilitates the interaction with ACE2. We have identified changes in bonding due to mutations in the RBM. The calculated bond order, based on AABPU, reveals that the Omicron mutations increase the binding strength of RBM to ACE2. Our findings correlate with and are instrumental to explain the current observations and can contribute to the prediction of next potential new variant of concern.

## 1. Introduction

COVID-19, caused by SARS-CoV-2, has adversely affected global health and economics for more than two years now. It has taken millions of lives and caused chaos in every aspect of our lives. There has been several experimental [1,2] and computational [3,4,5,6] studies for effective vaccines and drugs. Despite significant progress in SARS-CoV-2 research yielding effective vaccines, the virus continues to mutate and develop, continually creating new variants of concern (VOCs) [7,8,9,10,11] and variants of interest (VOIs) [12,13,14,15,16] that have instigated new anxieties. The newly emerging variants can change virus properties such as transmissibility, antigenicity, infectivity, and pathogenicity [17]. The Omicron variant (OV) is the most recently identified SARS-CoV-2 VOC that has a substantially larger number of mutations and a much higher rate of transmission than previous variants [18,19,20]. The first case of OV was reported to World Health Organization (WHO) from South Africa on 24 November 2021 [21]. Ever since, the OV has been rapidly spreading all over the world and has become the dominant variant. In fact, the OV has been estimated to account for 99.5% of all cases in the US by the Center for Disease Control and Prevention (CDC) [22]. The transmissibility and severity of this variant, as well as its ability to evade vaccines and cause reinfections, remain unknown. These developments instigated a strong research focus to identify the impact of OV on the efficacy of vaccines and to improve/develop drugs to mitigate its effects. Hence, elucidating the effects of OV mutations on the binding with human cells is necessary to provide a solid understanding of the molecular basis of this variant.

OV has more than 30 mutations in the spike (S) protein, including 15 mutations in its receptor binding domain (RBD) [23]. The S protein of SARS-CoV-2 is responsible for the viral entry into the human cell and activating the infection, in particular since the RBD attaches directly to human angiotensin-converting enzyme 2 (ACE2). Therefore, the S protein and its RBD are considered targets for vaccination and therapeutic development [3,24,25,26,27]. The receptor binding motif (RBM) in the RBD is the main functional motif that forms the interface between the S protein and ACE2 [28,29,30]. Among the 15 mutations in RBD, the RBM consists of 10 mutations: N440K, G446S, S477N, T478K, E484A, Q493R, G496S, Q498R, N501Y, and Y505H [20]. This unusually large number of OV mutations has promoted valid concerns about their impact on the efficacy of existing vaccines and treatments [31,32,33,34]. More specifically, these concerns became further exacerbated when it was discovered that some OV mutations at the RBM, such as T478K, E484A, and N501Y, which have also been found in previous variants [35], have been demonstrated to influence ACE2 binding or have been involved in escaping antibodies [35,36,37]. On the other hand, the other seven mutations of RBM are unique to OV, and their biological functions are still undetermined.

In this context, the investigation of how these OV mutations interact with the ACE2 human receptor is crucial for understanding the efficiency of viral entry and the ensuing speed of viral proliferation. Moreover, it could also provide important information regarding the identification of significant epitopes on RBM to guide the therapeutic development of efforts to counter SARS-CoV-2 variants. In this work, we focus on the 10 mutations in RBM of OV and the changes they instigate in the interaction of amino acids (AAs) at the interface of RBM and ACE2, between the unmutated or wild-type (WT) and mutated OV virus types. Ab initio quantum mechanical calculation based on density functional theory (DFT) has been implemented to gain deep insights into these interactions at the atomic as well as the AA scale. A novel concept of amino acid–amino acid bond pair unit (AABPU) has been developed to emphasize the changes in the bonding and other properties such as shape, volume, surface, and their partial charge.

## 2. Model Constructions

**Figure 1:**
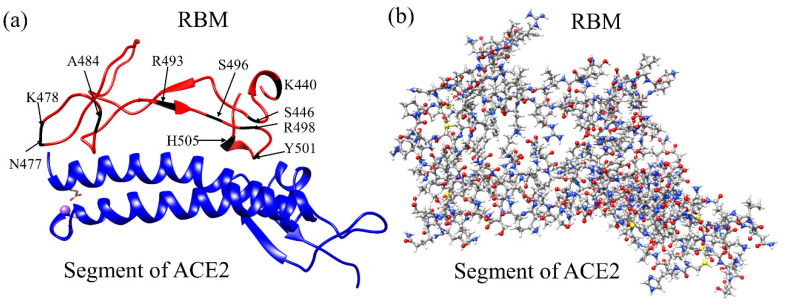
The RBM-ACE2 interface model. (**a**) Ribbon structure showing the interface between the RBM and segment of ACE2. Ten mutated AAs of the RBM in OV are marked. (**b**) The ball and stick structure of the same model in (**a**): Grey: C; red: O, blue: N; and white: H. In the WT interface model, there are 1102 atoms in RBM, 1822 atoms in ACE2 segment, and 6 Na^+^ ions, with a total of 2930 atoms. In OV interface model, there are 1141 atoms in RBM, 1822 atoms in ACE2 segment, and 1 Na^+^ ions with a total of 2964 atoms.

The present work focuses on the interactions in the interface complex between RBM and a portion of ACE2 derived from the PDB ID 6M0J [29], as detailed in our previous publication [38]. This system contains 71 amino acids (AAs) from S438 to Y508 of the RBM and 117 AAs from S19 to I88 and G319 to T365 of the ACE2. To neutralize the model, we have also added 6 Na^+^ ions and a single Na^+^ ion to unmutated or the wild-type (WT) model and Omicron mutated model, respectively. The placing of the Na^+^ ions was performed via a Coulomb potential on a grid, using the LEaP program in the AMBER (Assisted Model Building with Energy Refinement) package. Hydrogen (H) atoms were added using the LEaP module [39,40]. We used the Dunbrack backbone-dependent rotamer library [41] implemented by UCSF Chimera [42] to mutate the 10 AAs for the Omicron variant. For torsion and angle adjustment of T478K and N501Y, we used data from PDB ID 7ORA [14] and 7V80 [43], respectively. The WT and OV model consist of a total of 2930 atoms and 2964 atoms, respectively, as illustrated in Figure 1. These two interface models (WT and OV) are fully optimized using the Vienna ab initio simulation package (VASP) [44], and then the optimized structure is used as input for electronic structure calculation using the orthogonalized linear combination of atomic orbitals (OLCAO) [45]. Details of these methods are discussed in Section S1 of Supplementary Material (SM). The combination of using these two different DFT codes is well documented and is especially effective for large complex biomolecular systems such as the S-protein [38,46,47,48,49].

## 3. Results

In OLCAO, the bond order (BO) or strength of bonds is calculated using Mulliken’s Scheme [50,51]. The traditional BO description has been extended to quantify the bonding strength between two AAs (u,ν) called amino acid-amino acid bond pair, AABP (u,v) [46] in Equation (1), since in biomolecular systems, the use of AABP is more useful than interatomic bonding between a pair of atoms.

(1) $$AABP\left(u,\;v\right)=\underset{\alpha \epsilon u}{\sum }\underset{\beta \epsilon v}{\sum }\rho_{\alpha i,\beta j}.$$

The AABP considers all possible bonding between two AAs, including both covalent and hydrogen bonding (HB). This single quantitative parameter, provided by the electronic structure, reflects the internal bonding strength between AAs. AABP can be further resolved into nearest neighbor (NN) pairs and non-local (NL) bonding from non-NN pairs along the protein backbone. We can further identify the contribution arising from HB to AABP. Hence, AABP is an ideal parameter to characterize interactions between different AAs or groups of interacting AAs in biomolecules. In order words, we can consider AABP to be the basic biological unit that we refer to as the AAPB unit (AABPU). This will be thoroughly elaborated in the following section.

### 3.1. Analysis of AABPU for Mutation in RBM-ACE2

**Figure 2:**
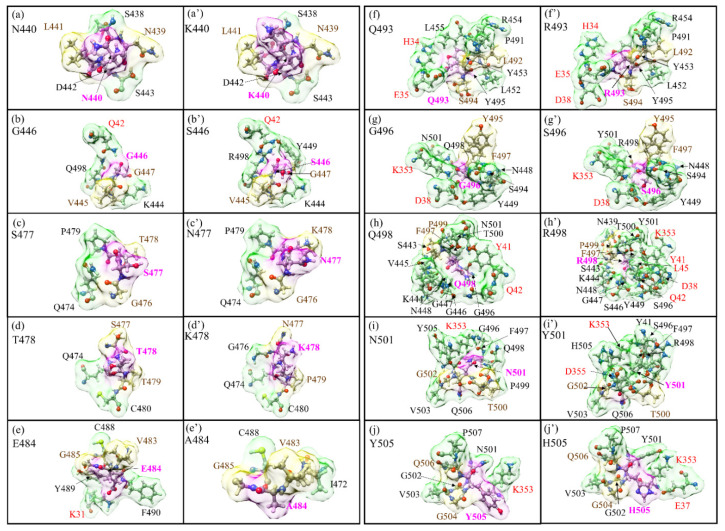
Details of the shape change of AABPU of the ten mutation sites in RBM: (**a**) N440, (**b**) G446, (**c**) S477, (**d**) T478, (**e**) E484, (**f**) Q493, (**g**) G496, (**h**) Q498, (**i**) N501, and (**j**) Y505 for the WT. (**a′**) K440, (**b′**) S446, (**c′**) N477, (**d′**) K478, (**e′**) A484, (**f′**) R493, (**g′**) S496, (**h′**) R498, (**i′**) Y501, and (**j′**) H505 for the OV. The surface of mutated sites is shown in magenta, and surfaces of NN and NL are shown in yellow and green, respectively. All NN and NL AAs are marked near to their surface in brown and black, respectively. NL AAs from ACE2 are marked in red.

In this section, we characterize the overall behavior of the ten mutations in the RBM–ACE2 interface model for the OV in terms of the key parameters in AABPU: total AABP, NN AABP, AABP due to NL interactions, AABP from HBs, number of NL AAs, the volume and surface area of the AABPU, and the calculated partial charge (PC) for the AABPU; see Table 1. Pairs of subsequent rows correspond to an unmutated WT site (light blue) and a mutated OV site (light orange), respectively. For example, the first two rows show the calculated key parameters for N440K mutation, wherein both WT and OV cases N440 and K440 interact with two NN AAs and 3NL AAs form two different AABPUs of six AAs each but with different volumes as well as values for PC. The change in shape for the 10 mutations in the RBM-ACE2 model is depicted in Figure 2.

In Figure 2, we show the change in the shape of the 10 AABPU for WT and OV, side by side, for easy comparison, with the details of bonding configurations not shown in Figure 2 listed in Tables S1–S20 in SI for WT and OV shown side by side. In these tables, the colors for the NN and NL bonds are in yellow and green, consistent with the colors used for the surface shown in Figure 2. Detailed inspection of Figure 2 reveals substantial changes in shapes, orientations, and number of NL AAs due to mutations. The most prominent is exhibited by the Q498 to R498 mutation, where the number of NL AAs has increased from 11 to 15 (Figure 2h,h′). On the other hand, for the mutation from E484 to A484, the number of NL AAs decreased from four to two (Figure 2e,e′). These changes will affect the overall bonding in the AABPU of the mutated sites.

The AABPU, as mentioned above, is an important aggregation of parameters to show the changes in the interaction due to mutation. There are several important points from Table 1 and Figure S1 that should be explicitly pointed out. (1) Mutation increases the total AABP in 6 out of 10 sites., i.e., G446S, S477N, T478K, Q493R, Q498R, and N501Y. (2) The change in total AABP is affected by both NN AABP and NL AABP. Specifically, mutation increases the NN AABP in 5 out of 10 sites (G446S, S477N, T478K, E484A, and Q493R) and increases the NL AABP in 5 out of 10 sites (G446S, T478K, Q498R, N501Y, and Y505H). Hence, mutation increases or decreases the overall bonding strength depending on the site of mutation. Each site is unique in terms of inter-amino acid interaction and behaves accordingly. (3) Mutation increases the contribution from HB to the total AABP in 6 out of 10 sites (G446S, S477N, T478K, Q493R, Q498R, and Y505H). (4) Mutation increases the number of NL AAs in 5 out of 10 sites (G446S, T478K, Q498R, N501Y, Y505H) but just by one. However, in site Q498R, the NL AAs increases by four and in E484A decreases by two. In fact, E484A is the only site where the number of interacting NL AAs decreases after mutation. (5) The increase in the number the of NL AAs seems to increase its total AABP. However, there could be an outlier such as in Y505H, which has one more NL AA, but its total AABP becomes lower after mutation due to a decrease in the NN AABP after mutation.

**Figure 3:**
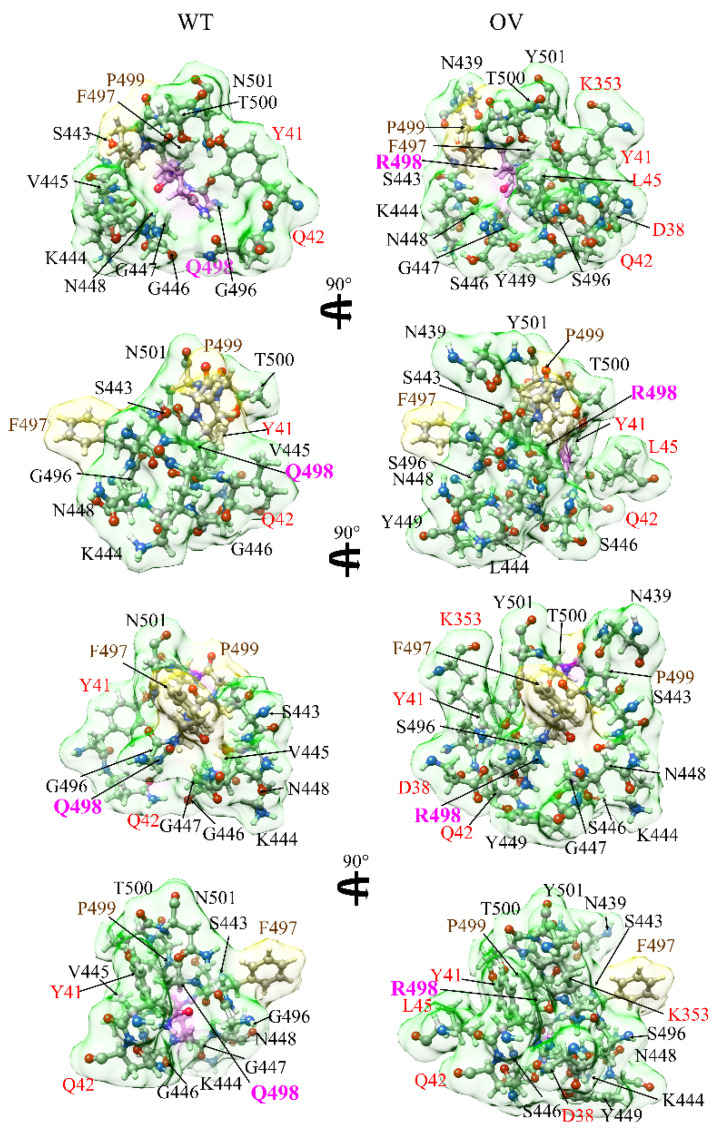
Interaction change due to mutation at site 498 for both WT (**left**) and OV (**right**). The surface of site 498 is marked in magenta color. The surfaces of its NN AAs and NL AAs are shown in yellow and green, respectively. Visible NN and NL AAs are marked near their surface in brown and black, respectively. NL AAs from ACE2 are marked in red. We can see considerable changes in NL AAs and their shapes due to mutation.

In analyzing AABPU, we focus on the change in volume and surface area of the unit. Mutation increases the volume in 8 out of 10 sites except in E484A and Q493R. In E484A, the number of interacting NL AAs decreases from four to two, the volume decreases by 45.6%, and the surface area decreases by 39.1%. In Q493R, The NL AAs are the same (8): the decrease in volume (1.0%) is negligible and the increase in surface area (19.4%) is relatively small. On the other hand, the most prominent changes occur in Q498R: the NL AAs increase from 11 to 15, the volume increases by 56.2%, and surface area increases by 34.0%. The changes in volume, surface area, and shape of AABPU due to the mutation provide an overall picture of change in the geometry and structure due to mutation, indicating the key role of the inter-AAs’ interaction based on interatomic bonding. In Figure 3, we show the details of changes in the volume and shape for mutation Q498R in four different orientations with the AAs in the unit marked. It seems obvious that the significant mutation-driven change in all aspects for this AABPU could be one of the reasons leading to the high infectivity of Omicron to be discussed in Section 4.

### 3.2. Electronic Structure and Bonding

Traditionally, the electronic structure in materials science and condensed matter physics is discussed in the context of the total density of states (TDOS) and the partial density of states (PDOS) of its different components. Figure S2 shows the TDOS for both WT and the OV from −25 eV to 25 eV. The overall features of the TDOS for WT and OV are very similar, since they contain similar atomic components. Both have a very low gap between the highest occupied molecular orbital (HOMO) and the lowest unoccupied molecular orbital (LUMO).

One of the advantages of the OLCAO method is in providing details of the strength of the bond between every pair of atoms involved in the system. In Figure S3a,b, we display the bond order (BO) vs. bond length (BL) for every pair of atoms in WT and OV of the RBM-ACE2 model, respectively. The atomic pairs with very short bond lengths, i.e., of 1 Å to 1.1 Å, are N-H, O-H, and C-H bonds. C-O bonds with 1.2 Å to 1.4 Å BL have the highest BO, which fluctuates from 0.63 e^−^ to 0.19 e^−^. In the similar range of BL of 1.3 Å to 1.5 Å, the N-C BO ranges from 0.57 e^−^ to 0.12 e^−^. Similarly, from 1.4 Å to 1.7 Å, C-C BL can be roughly separated into two groups, one with higher BO from 0.65 e^−^ to 0.54 e^−^ and the other with lower BO, with the higher BO pair stemming from the double bonds and those with lower BO from the single bonds. There are also notable C-S bonds, with S (sulfur) from AAs such as Cys, Met, and His. We can see a significant amount of HBs in the form of N⋯H and O⋯H, with much lower BO and larger BL. There are a few O-Na bonds at BL from 2 Å to 2.3 Å.

While the overall bonding between WT and OV looks similar, there are some differences; for example, WT has 19 types of different bonds, whereas OV has 18 types, with N-Na bonds completely missing in OV. We also notice a significantly stronger C-Na bond between C from I88 and Na ion in the OV case. In the inset for Figure S3a,b, which shows the much smaller BO values at the larger BL from 2.5 Å to 4.5 Å, we notice a significant number of C-C, C-H, N-C, H-H, and HBs. Even though these bonds have lower BO, their large number makes them significant. The differences between WT in (a) and OV in (b) are present but difficult to be clearly delineated at atomic scale.

### 3.3. Interaction of RBM with ACE2

**Figure 4:**
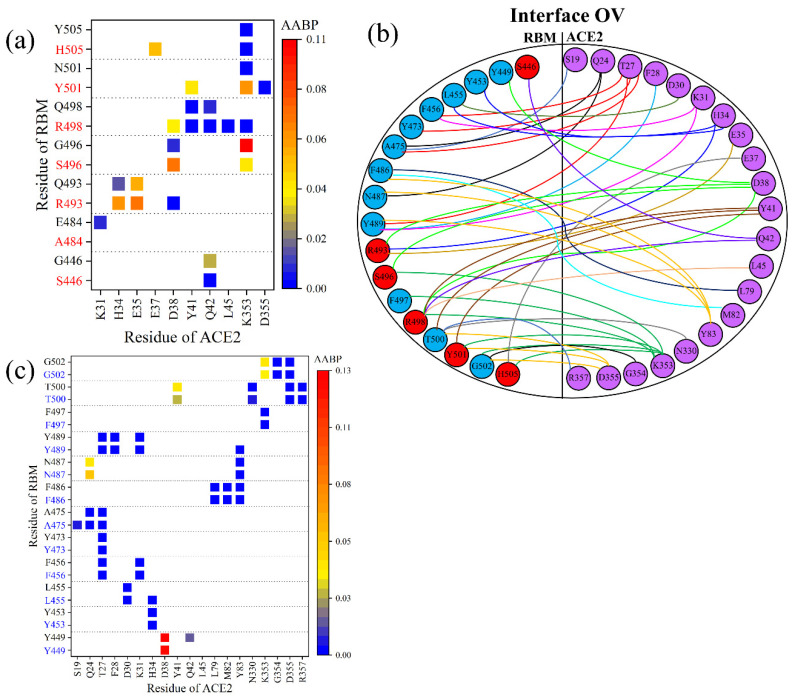
Comparison of RBM–ACE2 interaction in the interface model for WT and OV. (**a**) AABP map focusing on changes in bonding at the interface of mutated sites for WT (black) and OV (red). (**b**) Interactions at the RBM-ACE2 interface for OV. Interface-interacting AAs in RBM are shown in blue and red circles, denoting mutated and unmutated AAs, respectively. Similarly, interacting AAs of ACE2 are shown in purple. Different colored lines are used just for clarity. (**c**) AABP map focusing on changes in bonding of interface of unmutated sites for WT (black) and OV (blue).

We now switch our focus to the interface between RBM and ACE2. Figure 4a summarizes the interface interaction between the mutated sites of RBM and ACE2. In the WT, 7 out of 10 AAs interact with the ACE2, while in the OV, only six mutated AAs (S446, R493, S496, R498, Y501, H505) interact with the ACE2. After the mutation in OV, A484 loses its bonding with the ACE2. There is a slight decrease in AABP values of S446 with ACE2, indicating a decrease in the interaction. Nevertheless, there are new bonding pairs formed by R493, R498, Y501, and H505, significantly increasing the strength in the RBM–ACE2 interaction. Y501, which has been seen in other mutations, is known to increase affinity with ACE2 [35]. Even though S496 interacts with the same components of ACE2, it has a slightly increased AABP value, also implying an increase in the strength of interaction with ACE2. Omicron RBM mutations are found in position, which have key contact with ACE2 such as R493 and S496 [52]. Based on the bonding with ACE2, we contend that these six mutated AAs just described can be more critical compared to the effect of the remaining mutated AAs, with five out of six mutated AAs engendering a higher AABP value in the bonding with ACE2, indicating an increased binding of RBM to ACE2.

Only the changes in the bonding of mutated AAs have been discussed so far, but there are also many other unmutated AAs in the RBM that interacts with ACE2, which should also be a point of scrutiny. It would thus be of interest to see if there are any changes in the interaction between the unmutated AAs in RBM with ACE2. Figure 4b shows the complicated topology of the interaction between RBM and ACE2 of the OV in its entirety. This sketch includes the 12 unmutated AAs (blue circles) in addition to 6 mutated AAs (red circles) in the RBM. Figure 4c shows a detailed AABP map for unmutated AAs for both WT and OV. Among the 12 unmutated AAs in OV, it is only the Y449 that has lost its bonding pair with ACE2, while T500 exhibits a reduction in AABP value with Y41 of ACE2. However, the three unmutated AAs (L455, A475, and Y489) have increased their bonding with ACE2, so the changes due to mutation also affect the bonding of unmutated AAs, which may also play its role in the increased transmissibility of the OV.

### 3.4. Partial Charge of AABPU

**Figure 5:**
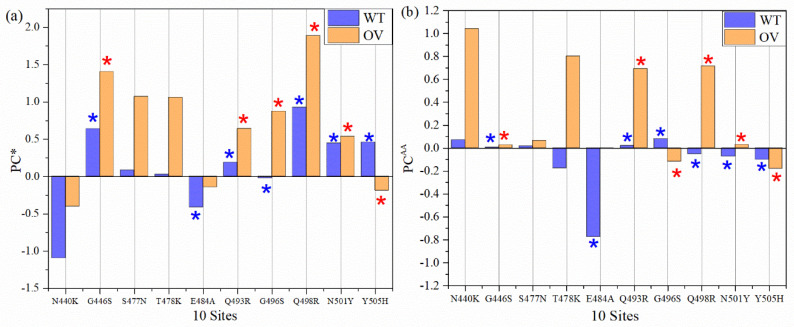
Partial charge (PC) for 10 sites before and after mutation: (**a**) PC per AABPU (PC*) for 10 sites (**b**) PC for each AA (PC^AA^). One out of ten mutated AABPU (Y505H) flips from positive PC* to negative PC* in relatively small amount. The PC* for mutation Q498R AABPU more than doubled. Two out of ten AAs (G496S and Y505H) PC^AA^ value increase in the negative direction. The blue and red (*) signs denote AAs in WT and OV, respectively, that interact with ACE2.

Partial charge (PC) is the most important parameter representing the electrostatic potential around a molecule and is instrumental in predicting the overall long-range intermolecular interaction [53]. We sum them up the calculated PC for each atom for all interacting AAs in AABPU for the ten mutations in RBM of OV in Table 1 and plotted in Figure 5a denoted by PC*. For example, the PC* for site N440 is −1.096 e^−^, obtained from the sum of PC of all interacting AAs N339, N440, L441, S438, D442, and S443. In the OV interface model, the PC* for the mutated K440 is −0.402 e^−^ by summing the PCs of N439, K440, L441, S438, D442, and S443. This is a significant increase toward the positive PC or equivalently a reduction in the negative PC for the mutation N440K. Figure 5a shows the comparison of PC* for the 10 sites AABPU before and after mutation. Interestingly, 9 out of 10 sites exhibit an increase in PC* to a more positive charge. The only site that changed from positive to negative PC is Y505H and even that one only slightly. Ostensibly, the biggest increase in the positive charge is in mutation Q498R from +0.926 e^−^ to 1.889 e^−^, which is more than double. Figure 5b shows the comparison of PC for each AA (PC^AA^). It shows 10 sites before and after mutation, with 8 out of 10 AAs exhibiting an increased PC into a more positive charge. Our findings align well with some other indications that PC resulting from mutations may increase [54]. While there have been other studies that have emphasized the dominance of positively charged AAs implicated in the increased electrostatic interactions [54,55,56], we provide the only quantitative estimate of the effect of the mutations on the PC derived from ab initio computation.

It must be emphasized that in Figure 5a, PC* stands for the AABPU, including also the interacting AAs of ACE2. Interestingly, even after interacting with AAs in ACE2, five out of six AABPUs still have a positive PC*, implying that after interacting with ACE2, the molecular units are more positively charged and may still have further interaction with the negatively charged AAs.

**Figure 6:**
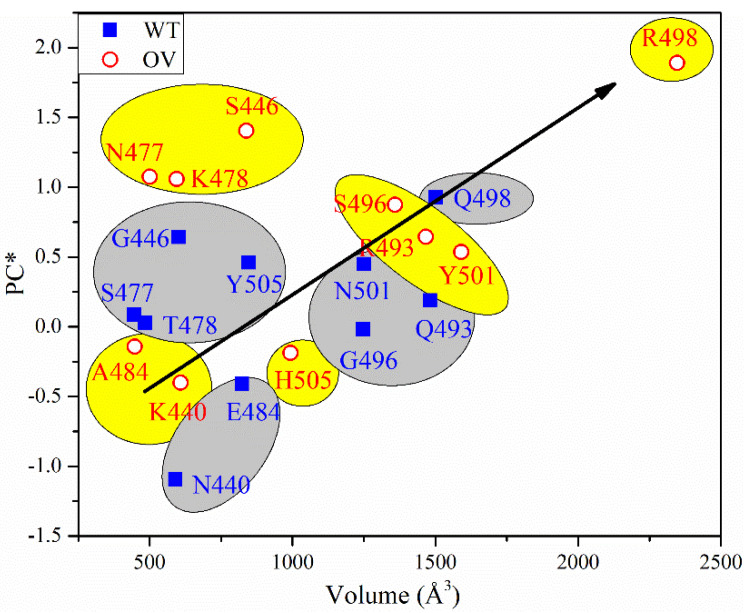
PC* vs. volume for 10 sites before and after mutation.

To sum up, all PC* for 10 sites of WT and OV give 1.245 e^−^ and 6.745 e^−^, respectively. The PC* from WT to OV shifts substantially towards a positive charge, with an impressive increase of 442%. This huge gain in PC* could be one of the major reasons for the rapid infectivity of OV, to be discussed further in Section 4. Finally, in Figure 6, we correlate the PC* of the 10 mutations with the volume of their AABPU. There is a clearly discernable general trend with mutations tending to make the PC more positive as well as increasing the volume of AABPU, especially and remarkably for the mutation of Q498R.

## 4. Source of High Infection Rate in the Omicron Variant

The Omicron variant is known for its enhanced infectivity. There are many unanswered questions to this observation, especially at the fundamental level. Based on our detailed analysis of the AABPU data for the 10 mutations of OV, focusing on the changes in their volume and surface areas due to their AAs’ interactions, we can provide some useful insights. The increase in the total AABP value of the mutated AAs implies increased interaction within the respective AABPU, whereas a decrease would also imply a decrease in the interaction and consequently an increase in the flexibility in the AABPU. The changes in the number of interacting NL AAs are also critical and will modify the interactions with other AAs, especially those in ACE2, possibly being one of the major reasons for the increased OV infectivity. Among the 10 mutated AAs in OV, 6 interact with ACE2 and 5 have an increased AABP value, indicating that the effect of the mutation is to increase the binding of RBM to ACE2. In addition, there is also an increase in AABP values between a few unmutated AAs of RBM with ACE2 as an effect of mutation.

Based on our calculated PC values for each AA, 8 out of 10 AAs exhibit an increase in the PC^AA^ towards positive charge, confirming the conspicuous dominance of the positively charged AAs as a result of mutations. In addition, our calculations also provided a deeper description of the changes in PC, including the interactions with ACE2. In fact, in 9 out of 10 sites in AABPU, the mutation enforces a positive PC*, which indicates that such AABPU will interact more strongly with negatively charged AAs. Moreover, volume increase in these AABPUs follows the increase in their PC*, respectively. The mutations described are mostly limited to the surface of RBM, and the overall positive PC* of these sites can thus be related to the high infection rate in the OV, consistent also with studies suggesting the development of specific antibodies with mostly negatively charged AAs for better binding [55,57], as well as the non-specific interactions with predominantly anionic biological membranes [58].

## 5. Conclusions

In summary, we have used the novel concept of AABPU as a basic biomolecular unit in complex proteins to provide detailed information on the effect of 10 mutations in RBM at the interface of RBM-ACE2. For the first time, we also provide accurate values of the volume, surface area, partial charge, and other parameters in AABPU at an atomic level obtained via detailed ab initio quantum chemical calculations. In particular, the effects of the most important and complicated OV mutation, Q498R, are clearly described and characterized. This comprehensive investigation of the ab initio atomic resolution of the Omicron variant sheds important light on the fundamental molecular logic behind its enhanced infectivity and paves the way towards a rapid analysis and characterization of the possible next variant(s) of concern in the SARS-CoV-2 virus.

## Figures and Tables

**Table 1:** Comparison of results between wild-type (WT) and Omicron variant (OV) for their amino acid–amino acid bond pair units (AABP units) in ten RBM sites. The two mutations in red have their volumes decreased after mutation.

Models	Total AABP	NN AABP	NL AABP	AABP from HB	# NL AAs	Volume (Å^3^)	Area (Å^2^)	PC* (e^−^)
WT N440	0.983	0.979	0.005	0.037	3	590.9	466.8	−1.096
OV K440	0.983	0.977	0.006	0.037	3	608.9	502.5	−0.402
WT G446	0.976	0.945	0.032	0.067	3	601.9	530.6	0.641
OV S446	1.073	1.013	0.060	0.116	4	838.8	704.2	1.404
WT S477	0.962	0.955	0.007	0.039	2	447.1	388.7	0.085
OV N477	1.178	1.177	0.001	0.170	2	500.9	441.9	1.074
WT T478	1.064	1.062	0.002	0.022	2	485	427.1	0.027
OV K478	1.258	1.255	0.003	0.156	3	594.4	507.9	1.058
WT E484	1.049	0.930	0.119	0.133	4	823	678.8	−0.411
OV A484	0.947	0.944	0.002	0.032	2	447.9	413.5	−0.142
WT Q493	1.197	0.969	0.229	0.241	8	1482	986.5	0.188
OV R493	1.263	1.058	0.205	0.270	8	1467	1070	0.644
WT G496	1.127	0.992	0.135	0.158	7	1247	929.2	−0.021
OV S496	1.098	0.964	0.134	0.146	7	1359	952.2	0.874
WT Q498	1.117	1.072	0.045	0.052	11	1502	1019	0.926
OV R498	1.220	1.058	0.162	0.147	15	2346	1365	1.889
WT N501	1.091	0.963	0.128	0.135	8	1251	883.8	0.447
OV Y501	1.139	0.957	0.183	0.135	9	1591	988.9	0.536
WT Y505	1.038	0.960	0.078	0.102	5	847.2	637.8	0.460
OV H505	1.020	0.934	0.086	0.107	6	993.5	720.4	−0.188

Nearest neighbor (NN); non-local (NL); number of (#); partial charge for AABPU (PC*).

## Data Availability

All data are listed in tables or presented in figures in main text or Supplementary Material.

## Supplementary Materials

The following supporting information can be downloaded at: https://www.mdpi.com/article/10.3390/ijms23052870/s1. Additional descriptions, figures and tables are provided in the Supplementary Material.
