# Supplementary material for: Mutations of Omicron Variant at the Interface of the Receptor Domain Motif and Human Angiotensin-Converting Enzyme-2
Source: Int J Mol Sci. 2022 Mar 6;23(5):2870. doi: 10.3390/ijms23052870 (PMC8911136; doi:10.3390/ijms23052870)
Supplement: Supplementary file 1 [file ijms-23-02870-s001.zip › ijms-1607722-supplementary.pdf]

Supplementary Materials for
Mutations of Omicron variant at the interface of the receptor domain motif and human angiotensin-converting enzyme-2

Puja Adhikari[1], Bahaa Jawad[1,2], Rudolf Podgornik[3,4,5], and Wai-Yim Ching[1]

1. Department of Physics and Astronomy, University of Missouri-Kansas City, Kansas City, Missouri, 64110 USA
2. Department of Applied Sciences, University of Technology, Baghdad 10066, Iraq
3. School of Physical Sciences and Kavli Institute of Theoretical Science, University of Chinese Academy of Sciences, Beijing 100049, China
4. CAS Key Laboratory of Soft Matter Physics, Institute of Physics, Chinese Academy of Sciences, Beijing 100090, China
5. Wenzhou Institute of the University of Chinese Academy of Sciences, Wenzhou 325000, Zhejiang, China.

Content:

S1. Methods

Figures:
Figure S1: Comparison of (a) Total AABP, (b) NN AABP, (c) NL AABP, (d) AABP from HB, and (e) No. of NL AAs for 10 unmutated (WT) and mutated (OV) AAs. (f) Volume, and (g) Surface for 10 sites or AABPU of RBM-ACE2 interface model for WT and OV.
Figure S2: TDOS of RBM-ACE2 interface model for WT and OV.
Figure S3: BO vs. BL of RBM-ACE2 interface model for WT and OV.

Tables:
Table S1: N440 with their bonding for WT interface model.
Table S2: K440 with their bonding for OV interface model.
Table S3: G446 with their bonding for WT interface model.
Table S4: S446 with their bonding for OV interface model.
Table S5: S477 with their bonding for WT interface model.
Table S6: N447 with their bonding for OV interface model.
Table S7: T478 with their bonding for WT interface model.
Table S8: K478 with their bonding for OV interface model.
Table S9: E484 with their bonding for WT interface model.
Table S10: A484 with their bonding for OV interface model.
Table S11: Q493 with their bonding for WT interface model.
Table S12: R493 with their bonding for OV interface model.
Table S13: G496 with their bonding for WT interface model.
Table S14: S496 with their bonding for OV interface model.

Table S15: Q498 with their bonding for WT interface model.
Table S16: R498 with their bonding for OV interface model.
Table S17: N501 with their bonding for WT interface model.
Table S18: Y501 with their bonding for OV interface model.
Table S19: Y505 with their bonding for WT interface model.
Table S20: H505 with their bonding for OV interface model.

References

S1. Methods:

S1.1 Vienna *ab initio* simulation package (VASP):

The two interface models are fully optimized by using Vienna *ab initio* simulation package (VASP) known for its efficiency in structure optimization [1]. We use the projector augmented wave (PAW) method with Perdew-Burke-Ernzerhof (PBE) exchange correlation functional [2] within the generalized gradient approximation (GGA). The input parameters used in VASP are as follows: energy cut-off 500 eV, electronic convergence of 10^{-4} eV, force convergence criteria for ionic steps at -10^{-2} eV/Å and a single k-point sampling. For the optimization, there is complete freedom for ionic position but not for cell volume, and cell shape. All VASP relaxations were carried out at the National Energy Research Scientific Computing (NERSC) facility at the Lawrence Berkeley Laboratory with special allocations and at the Research Computing Support Services (RCSS) of the University of Missouri System. The computational resources used for the structural relaxation are quite substantial because of the high accuracy required in the final structure and the slow convergence for the large complex biomolecular systems.

S1.2 Orthogonalized linear combination of atomic orbitals (OLCAO):

In house developed orthogonalized linear combination of atomic orbitals (OLCAO) method [3] is used for the electronic structure and interatomic interactions of the two interface models. Using the OLCAO method we calculate the effective charge (Q^*) on each atom and the bond order (BO) values $\rho_{\alpha\beta}$ between any pairs of atoms. They are obtained from the *ab initio* wave functions with atomic basis expansion:

$$Q_\alpha^* = \sum_i \sum_{m,occ} \sum_{j,\beta} C_{i\alpha}^{*m} C_{j\beta}^m S_{i\alpha,j\beta} \quad (1)$$

$$\rho_{\alpha\beta} = \sum_{m,occ} \sum_{i,j} C_{i\alpha}^{*m} C_{j\beta}^m S_{i\alpha,j\beta}. \quad (2)$$

In the above equations, $S_{i\alpha,j\beta}$ are the overlap integrals between the ith orbital in α^{th} atom and the jth orbital in the βth atom. $C_{j\beta}^m$ are the eigenvector coefficients of the m^{th}occupied molecular orbital level. The partial charge (PC) or ($\Delta Q_\alpha = Q_\alpha^0 - Q_\alpha^*$) is the deviation of the effective charge Q_α^* from the neutral atomic charge Q_α^0 on the same atom α. The BO quantifies the strength of the bond between two atoms and usually scales with the bond length (BL). The BL should be more accurately interpreted as the distance of separation of the two atoms since the BO value is influenced by the surrounding atoms. The calculation of PC and BO are based on the Mulliken scheme [4, 5].

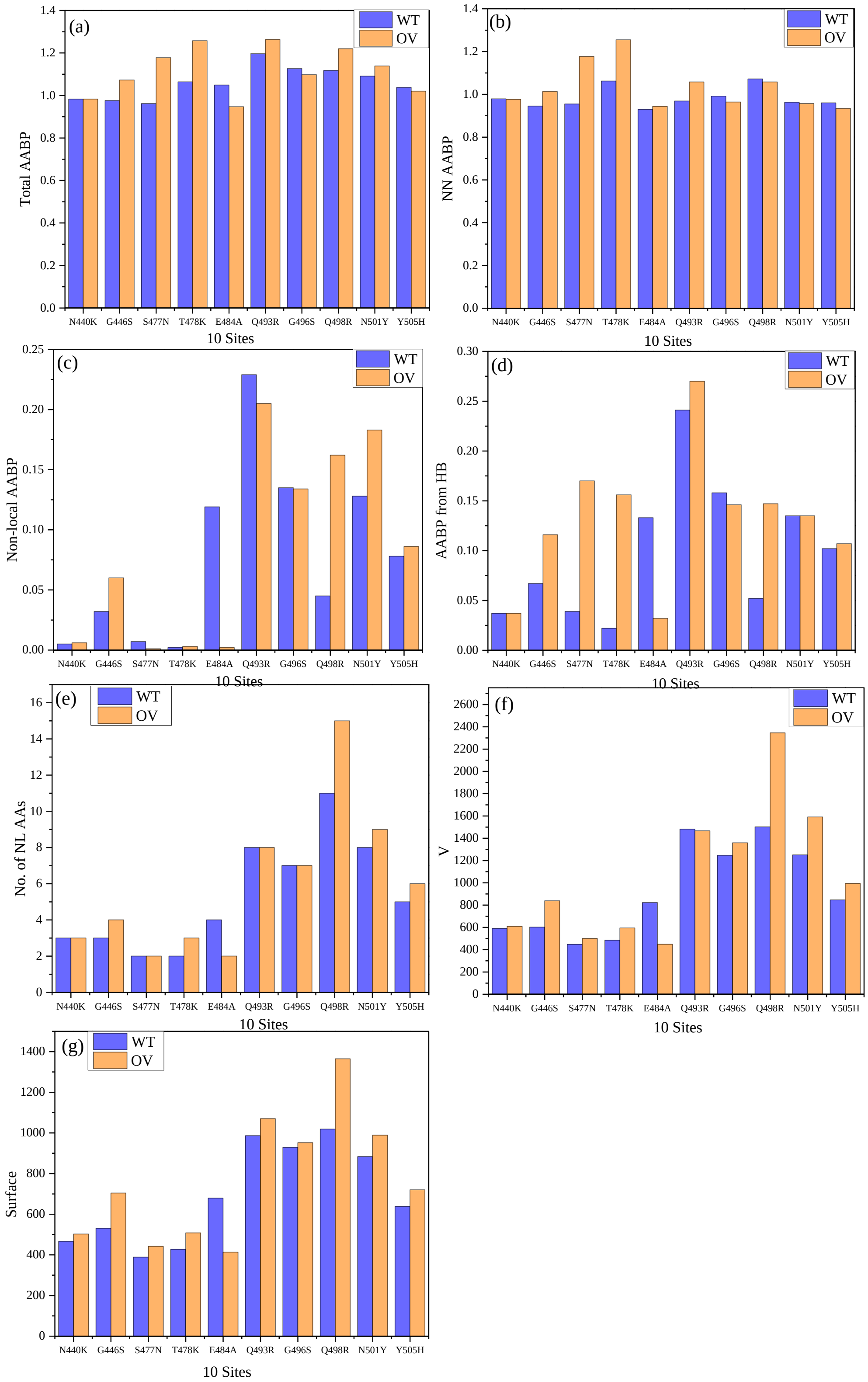

Figure S1: Comparison of (a) Total AABP, (b) NN AABP, (c) NL AABP, (d) AABP from HB, and (e) No. of NL AAs for 10 unmutated (WT) and mutated (OV) AAs. (f) Volume, and (g) Surface for 10 sites or AABPU of RBM-ACE2 interface model for WT and OV.

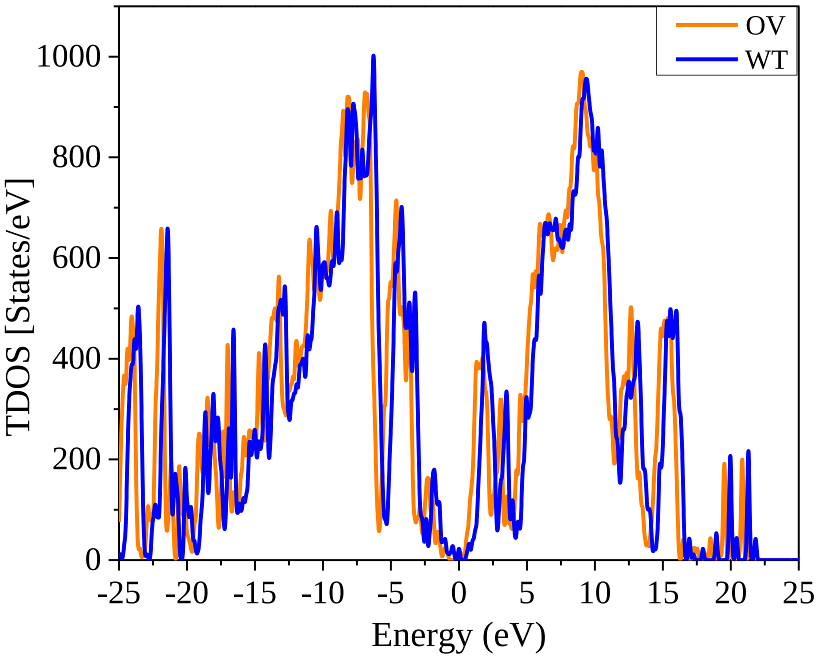

Figure S2: TDOS of RBM-ACE2 interface model for WT and OV.

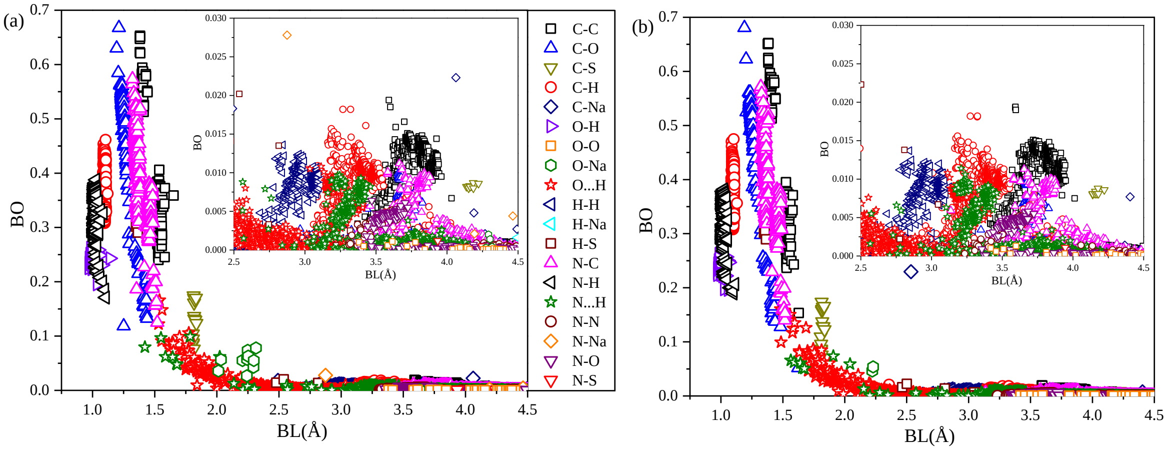

Figure S3: BO vs. BL of RBM-ACE2 interface model for **(a)** WT and **(b)** OV.

Table S1: N440 with their bonding for WT interface model.

Bond	BL	BO	AA1	AA2
N440				
C-C	3.555	0.0047	N440:C	L441:C
C-C	3.558	0.0074	N440:C	L441:CB
C-C	3.855	0.0128	N440:CA	L441:CA
C-O	3.426	0.0008	N440:C	N439:O
C-O	4.183	0.0006	N440:CB	N439:O
H-C	3.798	0.0011	N440:HB3	N439:C
H-C	4.071	0.0010	N440:HA	N439:CA
H-H	3.314	0.0010	N440:HA	L441:H
N...H	3.276	0.0048	N440:N	N439:HA
N-C	1.356	0.4557	N440:N	N439:C
N-C	4.191	0.0002	N440:N	N439:CG
N-C	4.235	0.0009	N440:N	L441:CA
O...H	2.427	0.0031	N440:HA	N439:O
O...H	3.193	0.0079	N440:H	N439:O
C-C	3.698	0.0122	N439:C	N440:CB
C-C	3.835	0.0133	N439:CA	N440:CA
C-C	4.324	0.0007	N439:C	N440:CG
C-O	4.118	0.0012	N439:C	N440:OD1
C-O	3.945	0.0010	L441:CB	N440:O
C-O	3.952	0.0002	L441:CG	N440:O
C-O	3.984	0.0007	L441:C	N440:O
H-C	3.752	0.0012	L441:H	N440:CB
H-C	4.037	0.0017	L441:HA	N440:CA
H-C	4.148	0.0002	L441:HG	N440:CA
H-H	3.578	0.0008	N439:HA	N440:H
N...H	3.111	0.0007	L441:N	N440:HA
N...H	3.926	0.0007	L441:N	N440:HB2
N-C	4.192	0.0009	N439:N	N440:CA
N-C	1.353	0.4199	L441:N	N440:C
N-C	3.576	0.0026	L441:N	N440:CB
O...H	2.372	0.0080	L441:HA	N440:O
O...H	2.812	0.0018	L441:HD21	N440:O
O...H	3.194	0.0087	L441:H	N440:O
O...H	4.314	0.0001	L441:HD22	N440:O
O...H	4.485	0.0001	L441:HD23	N440:O
C-O	4.060	0.0011	N440:C	S438:O
C-O	4.190	0.0005	N440:CA	S438:O
N...H	4.066	0.0001	N440:N	S438:HA
N...H	4.046	0.0014	N440:N	S443:HG
C-C	4.352	0.0002	S438:C	N440:CA
H-C	3.683	0.0010	D442:H	N440:C
H-C	4.305	0.0002	D442:H	N440:CA
N-C	3.962	0.0002	D442:N	N440:C

Table S2: K440 with their bonding for OV interface model.

Bond	BL	BO	AA1	AA2
K440				
C-C	3.543	0.0062	K440:C	L441:CB
C-C	3.598	0.0056	K440:C	L441:C
C-C	3.870	0.0126	K440:CA	L441:CA
C-O	3.493	0.0010	K440:C	N439:O
C-O	4.209	0.0004	K440:CB	N439:O
H-C	3.799	0.0010	K440:HB3	N439:C
H-C	4.064	0.0005	K440:HG2	N439:C
H-C	4.090	0.0010	K440:HA	N439:CA
H-C	4.137	0.0003	K440:HG3	L441:CA
H-C	4.242	0.0003	K440:HG3	L441:CB
H-C	4.373	0.0002	K440:HG3	L441:CD1
H-H	3.300	0.0009	K440:HA	L441:H
N...H	3.262	0.0047	K440:N	N439:HA
N-C	1.358	0.4492	K440:N	N439:C
N-C	4.186	0.0002	K440:N	N439:CG
N-C	4.251	0.0008	K440:N	L441:CA
O...H	2.465	0.0034	K440:HA	N439:O
O...H	3.150	0.0087	K440:H	N439:O
C-C	3.711	0.0121	N439:C	K440:CB
C-C	3.847	0.0131	N439:CA	K440:CA
C-C	4.422	0.0003	N439:C	K440:CG
C-O	3.895	0.0001	L441:CG	K440:O
C-O	3.923	0.0012	L441:CB	K440:O
C-O	4.052	0.0006	L441:C	K440:O
H-C	3.774	0.0012	L441:H	K440:CB
H-C	4.051	0.0004	L441:HG	K440:CA
H-C	4.059	0.0015	L441:HA	K440:CA
H-C	4.214	0.0002	L441:HG	K440:CB
H-C	4.406	0.0003	L441:HG	K440:CD
H-H	3.462	0.0012	N439:HA	K440:H
N...H	3.109	0.0002	L441:N	K440:HA
N...H	3.884	0.0001	L441:N	K440:HG2
N...H	3.942	0.0007	L441:N	K440:HB2
N-C	1.354	0.4248	L441:N	K440:C
N-C	3.592	0.0030	L441:N	K440:CB
N-C	4.190	0.0010	N439:N	K440:CA
O...H	2.406	0.0069	L441:HA	K440:O
O...H	2.784	0.0019	L441:HD21	K440:O
O...H	3.196	0.0089	L441:H	K440:O
O...H	4.243	0.0001	L441:HD22	K440:O
O...H	4.475	0.0001	L441:HD23	K440:O
C-O	3.928	0.0016	K440:C	S438:O
C-O	4.059	0.0006	K440:CA	S438:O
N...H	4.053	0.0014	K440:N	S443:HG
N...H	4.134	0.0001	K440:N	S438:HA
C-C	4.328	0.0003	S438:C	K440:CA
H-C	3.764	0.0011	D442:H	K440:C
H-C	4.414	0.0002	D442:H	K440:CA
N-C	4.045	0.0003	D442:N	K440:C

Table S3: G446 with their bonding for WT interface model.

G446				
C-C	3.457	0.0018	G446:C	G447:C
C-C	3.837	0.0135	G446:CA	G447:CA
C-O	3.762	0.0009	G446:C	V445:O
C-O	3.588	0.0002	G446:C	G447:O
H-C	3.279	0.0080	G446:HA2	V445:C
H-C	3.560	0.0013	G446:H	V445:CB
H-C	4.086	0.0015	G446:HA3	V445:CA
H-C	4.405	0.0001	G446:H	G447:CA
H-H	3.163	0.0004	G446:HA3	G447:H
H-H	3.521	0.0006	G446:HA2	G447:H
N...H	3.916	0.0006	G446:N	V445:HB
N...H	4.471	0.0002	G446:N	V445:HG12
N-C	1.361	0.3932	G446:N	V445:C
N-C	3.463	0.0012	G446:N	V445:CB
N-C	4.248	0.0006	G446:N	G447:CA
O...H	2.447	0.0075	G446:HA3	V445:O
O...H	3.175	0.0078	G446:H	V445:O
O...H	3.750	0.0001	G446:HA2	V445:O
C-C	3.469	0.0017	V445:C	G446:C
C-C	3.837	0.0137	V445:CA	G446:CA
C-O	3.771	0.0004	G447:C	G446:O
H-C	3.945	0.0012	V445:HA	G446:CA
H-C	3.211	0.0075	G447:HA2	G446:C
H-C	4.033	0.0018	G447:HA3	G446:CA
N...H	3.668	0.0012	V445:N	G446:H
N...H	3.261	0.0030	G447:N	G446:HA2
N-C	1.354	0.4523	G447:N	G446:C
N-N	3.501	0.0043	V445:N	G446:N
O...H	2.372	0.0095	G447:HA3	G446:O
O...H	3.194	0.0079	G447:H	G446:O
O...H	3.663	0.0005	G447:HA2	G446:O
C-O	4.105	0.0008	G446:C	K444:O
C-O	4.196	0.0005	G446:CA	K444:O
H-C	3.909	0.0001	G446:H	K444:C
O...H	3.314	0.0003	G446:H	K444:O
N-C	3.766	0.0007	G446:N	K444:C
H-C	4.450	0.0001	G446:H	Q498:CD
C-O	4.019	0.0009	Q42:CD	G446:O
H-C	4.392	0.0002	Q42:HE22	G446:C
H-H	4.317	0.0001	Q42:HE21	G446:HA3
O...H	1.997	0.0279	Q42:HE21	G446:O
O...H	3.362	0.0002	Q42:HE22	G446:O
H-C	4.061	0.0001	Q498:HG3	G446:CA

Table S4: S446 with their bonding for OV interface model.

S446				
C-C	3.492	0.0021	S446:C	G447:C
C-C	3.844	0.0133	S446:CA	G447:CA
C-O	3.631	0.0005	S446:C	G447:O
C-O	3.906	0.0012	S446:C	V445:O
H-C	3.310	0.0081	S446:HA	V445:C
H-C	3.438	0.0014	S446:H	V445:CB
H-C	3.941	0.0022	S446:HG	V445:CA
H-C	4.413	0.0001	S446:H	G447:CA
H-H	3.316	0.0003	S446:HB2	G447:H
H-H	3.498	0.0007	S446:HA	G447:H
N...H	3.861	0.0006	S446:N	V445:HB
N...H	4.418	0.0002	S446:N	V445:HG12
N-C	1.359	0.4278	S446:N	V445:C
N-C	3.432	0.0007	S446:N	V445:CB
N-C	4.201	0.0010	S446:N	G447:CA
O...H	2.081	0.0299	S446:HG	V445:O
O...H	3.165	0.0084	S446:H	V445:O
O...H	3.957	0.0009	S446:HB3	V445:O
C-C	3.498	0.0008	V445:C	S446:C
C-C	3.875	0.0132	V445:CA	S446:CA
C-O	3.860	0.0008	G447:C	S446:O
H-C	3.209	0.0074	G447:HA2	S446:C
H-C	3.558	0.0005	G447:H	S446:CB
H-C	3.941	0.0012	V445:HA	S446:CA
H-C	4.072	0.0015	G447:HA3	S446:CA
H-C	4.113	0.0001	V445:HG13	S446:CB
H-H	4.316	0.0001	V445:HG12	S446:H
N...H	3.250	0.0034	G447:N	S446:HA
N...H	3.663	0.0014	V445:N	S446:H
N...H	3.823	0.0004	G447:N	S446:HB3
N-C	1.354	0.4615	G447:N	S446:C
N-N	3.505	0.0043	V445:N	S446:N
O...H	2.409	0.0083	G447:HA3	S446:O
O...H	3.200	0.0080	G447:H	S446:O
O...H	3.650	0.0004	G447:HA2	S446:O
C-C	4.080	0.0010	S446:C	R498:CZ
C-O	4.075	0.0010	S446:C	K444:O
C-O	4.248	0.0005	S446:CA	K444:O
H-C	3.967	0.0002	S446:H	K444:C
O...H	3.412	0.0003	S446:H	K444:O
N-C	3.795	0.0007	S446:N	K444:C
C-O	3.628	0.0007	R498:CZ	S446:O
H-C	3.986	0.0016	R498:HH21	S446:CA
H-C	4.024	0.0001	Y449:HE2	S446:C
H-C	4.309	0.0001	R498:HG3	S446:CA
H-C	4.497	0.0002	R498:HH22	S446:C
O...H	1.867	0.0525	R498:HH21	S446:O
O...H	3.029	0.0002	Q42:HE21	S446:O
O...H	3.537	0.0008	R498:HH22	S446:O
N-C	3.695	0.0004	R498:NH2	S446:C

Table S5: S477 with their bonding for WT interface model.

S477				
C-C	3.653	0.0124	S477:C	T478:CB
C-C	3.856	0.0134	S477:CA	T478:CA
C-O	3.664	0.0004	S477:C	G476:O
C-O	4.124	0.0007	S477:CB	G476:O
C-O	4.059	0.0002	S477:C	T478:O
C-O	4.069	0.0004	S477:C	T478:OG1
H-C	3.690	0.0011	S477:HB3	G476:C
H-C	4.038	0.0014	S477:HA	G476:CA
H-C	4.478	0.0002	S477:H	T478:CA
H-H	3.339	0.0007	S477:HA	T478:H
N...H	3.848	0.0010	S477:N	G476:H
N-C	1.364	0.4301	S477:N	G476:C
N-C	4.219	0.0009	S477:N	T478:CA
O...H	2.424	0.0058	S477:HA	G476:O
O...H	3.186	0.0078	S477:H	G476:O
C-C	3.462	0.0018	G476:C	S477:C
C-C	3.621	0.0109	G476:C	S477:CB
C-C	3.825	0.0126	G476:CA	S477:CA
C-O	4.065	0.0004	G476:C	S477:OG
C-O	3.674	0.0010	T478:C	S477:O
C-O	4.234	0.0004	T478:CB	S477:O
C-O	4.493	0.0001	T478:CG2	S477:O
H-C	3.997	0.0010	G476:HA3	S477:CA
H-C	3.729	0.0014	T478:H	S477:CB
H-C	4.107	0.0012	T478:HA	S477:CA
H-C	4.155	0.0001	T478:HG21	S477:CA
H-C	4.215	0.0002	T478:HG23	S477:C
H-C	4.219	0.0001	T478:HG1	S477:C
H-H	3.009	0.0006	G476:HA2	S477:H
N...H	3.883	0.0011	G476:N	S477:H
N...H	3.140	0.0008	T478:N	S477:HA
N...H	3.808	0.0011	T478:N	S477:HB2
N-C	1.347	0.4227	T478:N	S477:C
N-C	3.533	0.0019	T478:N	S477:CB
N-N	3.625	0.0043	G476:N	S477:N
O...H	2.538	0.0063	T478:HA	S477:O
O...H	3.192	0.0084	T478:H	S477:O
N...H	4.432	0.0001	S477:N	Q474:HG2
N-N	4.455	0.0003	Q474:NE2	S477:N
O...H	2.561	0.0063	P479:HD3	S477:O
O...H	4.173	0.0001	P479:HD2	S477:O
O...H	4.341	0.0001	P479:HG3	S477:O
N-C	4.036	0.0003	P479:N	S477:C

Table S6: N447 with their bonding for OV interface model.

N477				
C-C	3.530	0.0042	N477:C	K478:CB
C-C	3.555	0.0087	N477:C	K478:C
C-C	3.792	0.0010	N477:C	K478:CG
C-C	3.842	0.0126	N477:CA	K478:CA
C-C	4.027	0.0010	N477:C	K478:CE
C-O	3.886	0.0002	N477:C	G476:O
C-O	3.899	0.0012	N477:CB	G476:O
C-O	4.036	0.0005	N477:C	K478:O
H-C	3.388	0.0004	N477:HB3	G476:C
H-C	3.974	0.0021	N477:HA	G476:CA
H-C	4.084	0.0001	N477:HA	K478:CA
N...H	3.828	0.0009	N477:N	G476:H
N-C	1.371	0.4295	N477:N	G476:C
N-C	4.328	0.0005	N477:N	K478:CA
N-N	4.167	0.0001	N477:ND2	K478:NZ
O...H	2.287	0.0104	N477:HA	G476:O
O...H	3.196	0.0075	N477:H	G476:O
O...H	3.717	0.0001	N477:HB3	G476:O
C-C	3.506	0.0043	G476:C	N477:CB
C-C	3.576	0.0074	G476:C	N477:C
C-C	3.819	0.0123	G476:CA	N477:CA
C-C	4.305	0.0002	G476:C	N477:CG
C-O	3.571	0.0014	K478:CE	N477:O
C-O	3.794	0.0006	K478:CB	N477:O
C-O	4.138	0.0004	K478:C	N477:O
C-O	4.252	0.0009	G476:C	N477:OD1
H-C	3.598	0.0028	K478:HZ1	N477:CA
H-C	3.788	0.0003	K478:HG3	N477:C
H-C	3.838	0.0002	K478:HB2	N477:C
H-C	3.994	0.0011	G476:HA3	N477:CA
H-C	4.036	0.0010	K478:HA	N477:CA
H-C	4.045	0.0006	K478:HZ3	N477:C
H-C	4.067	0.0011	K478:H	N477:CB
H-C	4.126	0.0004	K478:HZ2	N477:C
H-C	4.269	0.0002	K478:HE2	N477:C
H-C	4.370	0.0003	K478:HD3	N477:C
H-H	3.086	0.0008	G476:HA2	N477:H
N...H	3.867	0.0011	G476:N	N477:H
N...H	4.057	0.0005	K478:N	N477:HB2
N-C	1.343	0.4977	K478:N	N477:C
N-C	3.543	0.0003	K478:NZ	N477:C
N-C	3.755	0.0062	K478:N	N477:CB
N-C	4.239	0.0004	K478:N	N477:CG
N-N	3.613	0.0041	G476:N	N477:N
O...H	1.596	0.1350	K478:HZ1	N477:O
O...H	2.458	0.0031	K478:HA	N477:O
O...H	2.723	0.0016	K478:HE2	N477:OD1
O...H	2.846	0.0015	K478:HD2	N477:OD1
O...H	3.199	0.0079	K478:H	N477:O
O...H	3.301	0.0001	K478:HZ2	N477:OD1
O...H	4.082	0.0001	K478:HD3	N477:OD1
O...H	4.302	0.0002	K478:HZ3	N477:OD1
O...H	4.348	0.0001	K478:HE3	N477:OD1
O...H	4.486	0.0001	K478:HD3	N477:O
H-C	3.945	0.0002	P479:HD3	N477:C
H-C	4.341	0.0001	Q474:HE22	N477:C
N-C	4.431	0.0001	P479:N	N477:C
N-C	4.464	0.0001	Q474:NE2	N477:CA

Table S7: T478 with their bonding for WT interface model.

T478				
C-C	3.638	0.0111	T478:C	P479:CG
C-C	3.662	0.0134	T478:C	P479:CB
C-C	3.857	0.0125	T478:CA	P479:CA
C-C	4.215	0.0014	T478:CB	P479:CD
C-C	4.405	0.0004	T478:CA	P479:C
C-C	4.421	0.0008	T478:CA	P479:CG
C-O	3.674	0.0010	T478:C	S477:O
C-O	4.234	0.0004	T478:CB	S477:O
C-O	4.493	0.0001	T478:CG2	S477:O
H-C	3.729	0.0014	T478:H	S477:CB
H-C	4.107	0.0012	T478:HA	S477:CA
H-C	4.155	0.0001	T478:HG21	S477:CA
H-C	4.215	0.0002	T478:HG23	S477:C
H-C	4.219	0.0001	T478:HG1	S477:C
H-C	4.044	0.0013	T478:HA	P479:CG
H-C	4.075	0.0005	T478:HA	P479:CA
H-C	4.090	0.0001	T478:HB	P479:CD
H-H	3.840	0.0001	T478:HB	P479:HD2
N...H	3.140	0.0008	T478:N	S477:HA
N...H	3.808	0.0011	T478:N	S477:HB2
N-C	1.347	0.4227	T478:N	S477:C
N-C	3.533	0.0019	T478:N	S477:CB
N-C	3.932	0.0020	T478:N	P479:CD
N-N	3.464	0.0017	T478:N	P479:N
O...H	2.538	0.0063	T478:HA	S477:O
O...H	3.192	0.0084	T478:H	S477:O
O...H	3.685	0.0001	T478:HB	P479:O
O...H	4.339	0.0001	T478:HG1	P479:O
C-C	3.653	0.0124	S477:C	T478:CB
C-C	3.856	0.0134	S477:CA	T478:CA
C-O	4.059	0.0002	S477:C	T478:O
C-O	4.069	0.0004	S477:C	T478:OG1
C-O	3.666	0.0091	P479:CD	T478:O
C-O	4.233	0.0010	P479:CB	T478:O
H-C	4.478	0.0002	S477:H	T478:CA
H-C	3.914	0.0005	P479:HG3	T478:C
H-C	4.163	0.0002	P479:HD3	T478:CB
H-C	4.171	0.0003	P479:HD2	T478:CB
H-H	3.339	0.0007	S477:HA	T478:H
N...H	3.425	0.0001	P479:N	T478:HB
N...H	3.766	0.0023	P479:N	T478:HG1
N-C	4.219	0.0009	S477:N	T478:CA
N-C	1.347	0.5251	P479:N	T478:C
N-C	3.569	0.0025	P479:N	T478:CB
O...H	2.769	0.0005	P479:HA	T478:O
O...H	3.961	0.0005	P479:HD3	T478:O
O...H	4.168	0.0002	P479:HD2	T478:O
C-O	4.091	0.0001	T478:C	Q474:OE1
H-C	4.490	0.0001	Q474:HG3	T478:CA
O...H	2.583	0.0007	Q474:HG3	T478:O
O...H	4.065	0.0002	Q474:HG2	T478:O
O...H	4.469	0.0001	Q474:HB2	T478:O
O...H	3.508	0.0002	C480:H	T478:O
O...H	3.996	0.0002	C480:HA	T478:O
N-C	4.023	0.0006	C480:N	T478:C

Table S8: K478 with their bonding for OV interface model.

K478				
C-C	3.619	0.0104	K478:C	P479:CG
C-C	3.679	0.0131	K478:C	P479:CB
C-C	3.858	0.0124	K478:CA	P479:CA
C-C	3.973	0.0014	K478:CB	P479:CD
C-C	4.401	0.0007	K478:CA	P479:CG
C-C	4.405	0.0002	K478:CA	P479:C
C-O	3.571	0.0014	K478:CE	N477:O
C-O	3.794	0.0006	K478:CB	N477:O
C-O	4.138	0.0004	K478:C	N477:O
H-C	3.598	0.0028	K478:HZ1	N477:CA
H-C	3.788	0.0003	K478:HG3	N477:C
H-C	3.838	0.0002	K478:HB2	N477:C
H-C	4.014	0.0013	K478:HA	P479:CG
H-C	4.036	0.0010	K478:HA	N477:CA
H-C	4.045	0.0006	K478:HZ3	N477:C
H-C	4.067	0.0011	K478:H	N477:CB
H-C	4.108	0.0006	K478:HA	P479:CA
H-C	4.126	0.0004	K478:HZ2	N477:C
H-C	4.269	0.0002	K478:HE2	N477:C
H-C	4.370	0.0003	K478:HD3	N477:C
H-H	4.030	0.0001	K478:HG3	P479:HD3
H-H	4.224	0.0001	K478:HA	P479:HG3
N...H	3.616	0.0007	K478:N	P479:HD3
N...H	4.057	0.0005	K478:N	N477:HB2
N-C	1.343	0.4977	K478:N	N477:C
N-C	3.543	0.0003	K478:NZ	N477:C
N-C	3.755	0.0062	K478:N	N477:CB
N-C	4.092	0.0017	K478:N	P479:CD
N-C	4.239	0.0004	K478:N	N477:CG
N-N	3.492	0.0027	K478:N	P479:N
O...H	1.596	0.1350	K478:HZ1	N477:O
O...H	2.458	0.0031	K478:HA	N477:O
O...H	2.723	0.0016	K478:HE2	N477:OD1
O...H	2.846	0.0015	K478:HD2	N477:OD1
O...H	3.199	0.0079	K478:H	N477:O
O...H	3.301	0.0001	K478:HZ2	N477:OD1
O...H	3.341	0.0001	K478:HB3	P479:O
O...H	4.082	0.0001	K478:HD3	N477:OD1
O...H	4.302	0.0002	K478:HZ3	N477:OD1
O...H	4.348	0.0001	K478:HE3	N477:OD1
O...H	4.486	0.0001	K478:HD3	N477:O
C-C	3.530	0.0042	N477:C	K478:CB
C-C	3.555	0.0087	N477:C	K478:C
C-C	3.792	0.0010	N477:C	K478:CG
C-C	3.842	0.0126	N477:CA	K478:CA
C-C	4.027	0.0010	N477:C	K478:CE
C-O	3.671	0.0092	P479:CD	K478:O
C-O	4.036	0.0005	N477:C	K478:O
C-O	4.261	0.0008	P479:CB	K478:O
H-C	3.846	0.0006	P479:HG3	K478:C
H-C	3.897	0.0004	P479:HD3	K478:CB
H-C	3.915	0.0003	P479:HD2	K478:CB
H-C	4.084	0.0001	N477:HA	K478:CA
H-C	4.198	0.0003	P479:HA	K478:CA
N...H	3.678	0.0015	P479:N	K478:H
N...H	3.922	0.0003	P479:N	K478:HB2
N-C	1.351	0.5016	P479:N	K478:C
N-C	4.328	0.0005	N477:N	K478:CA
N-N	4.167	0.0001	N477:ND2	K478:NZ
O...H	2.663	0.0007	P479:HA	K478:O
O...H	3.965	0.0005	P479:HD3	K478:O
O...H	4.202	0.0001	P479:HD2	K478:O
N-O	3.912	0.0001	K478:N	G476:O
C-C	4.318	0.0002	Q474:CG	K478:C
C-O	4.474	0.0004	Q474:CB	K478:O
H-C	4.174	0.0001	C480:H	K478:C
N-C	4.091	0.0005	C480:N	K478:C
O...H	2.458	0.0016	Q474:HG3	K478:O
O...H	3.687	0.0001	C480:H	K478:O
O...H	3.906	0.0002	Q474:HG2	K478:O
O...H	4.353	0.0001	C480:HA	K478:O
O...H	4.378	0.0001	Q474:HB2	K478:O

Table S9: E484 with their bonding for WT interface model.

E484				
C-C	3.721	0.0139	E484:C	G485:C
C-C	3.796	0.0143	E484:CA	G485:CA
C-O	3.512	0.0013	E484:C	V483:O
C-O	4.263	0.0003	E484:CB	V483:O
H-C	3.684	0.0005	E484:H	V483:CG2
H-C	4.010	0.0004	E484:H	V483:CG1
H-C	4.044	0.0005	E484:HB2	V483:C
H-C	4.123	0.0009	E484:HA	V483:CA
H-C	3.977	0.0010	E484:HA	G485:CA
N...H	4.081	0.0006	E484:N	V483:H
N...H	3.901	0.0011	E484:N	G485:H
N-C	1.365	0.4205	E484:N	V483:C
N-C	3.718	0.0002	E484:N	V483:CG2
N-C	4.440	0.0002	E484:N	V483:CG1
N-N	3.633	0.0052	E484:N	G485:N
O...H	2.503	0.0036	E484:HA	V483:O
O...H	3.170	0.0082	E484:H	V483:O
C-C	3.762	0.0122	V483:C	E484:CB
C-C	3.836	0.0124	V483:CA	E484:CA
C-C	4.363	0.0002	V483:CB	E484:CA
C-O	3.563	0.0001	V483:C	E484:O
C-O	4.256	0.0004	G485:C	E484:O
H-C	3.908	0.0004	V483:HB	E484:CA
H-C	4.118	0.0006	V483:HA	E484:CA
H-C	4.139	0.0005	G485:HA2	E484:CA
H-C	4.191	0.0004	G485:HA3	E484:CA
H-C	4.453	0.0002	G485:HA2	E484:CB
H-H	3.428	0.0001	V483:HG21	E484:H
H-H	4.017	0.0001	V483:HG13	E484:H
N...H	3.956	0.0009	V483:N	E484:H
N...H	3.759	0.0007	G485:N	E484:HB2
N...H	4.175	0.0001	G485:N	E484:H
N-C	1.359	0.4099	G485:N	E484:C
N-N	3.687	0.0050	V483:N	E484:N
O...H	3.454	0.0003	V483:HB	E484:O
O...H	2.731	0.0016	G485:HA3	E484:O
O...H	2.772	0.0024	G485:HA2	E484:O
O...H	3.200	0.0084	G485:H	E484:O
C-C	4.373	0.0004	E484:CD	Y489:C
C-C	4.486	0.0003	E484:CD	F490:CA
C-O	3.637	0.0011	E484:C	C488:O
H-C	4.054	0.0001	E484:HA	C488:CA
H-C	4.487	0.0001	E484:HB3	C488:C
H-C	4.474	0.0003	E484:HG2	Y489:CB
H-C	3.988	0.0001	E484:HG2	F490:CA
H-H	3.996	0.0002	E484:HG3	Y489:HA
H-H	3.226	0.0002	E484:HG3	F490:H
O...H	2.483	0.0012	E484:HA	C488:O
C-O	3.555	0.0025	Y489:C	E484:OE1
C-O	3.543	0.0036	F490:CA	E484:OE1
H-C	4.083	0.0001	F490:HB3	E484:CD
H-C	4.292	0.0006	F490:H	E484:CB
N-C	3.571	0.0041	F490:N	E484:CD
O...H	2.581	0.0080	K31:HZ2	E484:OE1
O...H	2.844	0.0018	K31:HE2	E484:OE1
O...H	3.275	0.0004	K31:HZ2	E484:OE2
O...H	4.035	0.0001	K31:HE3	E484:OE1
O...H	2.762	0.0001	Y489:HA	E484:OE1
O...H	1.557	0.0909	F490:H	E484:OE1
O...H	3.709	0.0028	F490:H	E484:OE2

Table S10: A484 with their bonding for OV interface model.

A484				
C-C	3.695	0.0138	A484:C	G485:C
C-C	3.817	0.0141	A484:CA	G485:CA
C-O	3.799	0.0010	A484:C	V483:O
C-O	4.046	0.0005	A484:C	G485:O
C-O	4.078	0.0006	A484:CB	V483:O
H-C	3.401	0.0001	A484:H	V483:CG2
H-C	3.648	0.0009	A484:HB3	V483:C
H-C	3.919	0.0006	A484:H	V483:CG1
H-C	4.016	0.0009	A484:HA	G485:CA
H-C	4.072	0.0014	A484:HA	V483:CA
O...H	2.412	0.0061	A484:HA	V483:O
O...H	3.165	0.0080	A484:H	V483:O
O...H	4.027	0.0001	A484:HB3	V483:O
N-C	1.362	0.4221	A484:N	V483:C
N-C	4.395	0.0003	A484:N	V483:CG1
N...H	3.921	0.0011	A484:N	G485:H
N...H	4.091	0.0005	A484:N	V483:H
N-N	3.623	0.0050	A484:N	G485:N
C-C	3.408	0.0015	V483:C	A484:C
C-C	3.634	0.0095	V483:C	A484:CB
C-C	3.828	0.0126	V483:CA	A484:CA
C-C	4.357	0.0006	V483:CB	A484:CA
C-O	3.707	0.0001	V483:C	A484:O
C-O	4.269	0.0003	G485:C	A484:O
H-C	3.922	0.0009	V483:HB	A484:CA
H-C	4.103	0.0004	V483:HA	A484:CA
H-C	4.108	0.0002	V483:HB	A484:C
H-C	4.147	0.0007	G485:HA3	A484:CA
H-C	4.254	0.0001	G485:HA2	A484:CA
H-H	3.982	0.0001	V483:HG22	A484:H
H-H	4.023	0.0001	V483:HG13	A484:H
H-H	4.412	0.0001	V483:HG12	A484:H
O...H	2.660	0.0026	G485:HA3	A484:O
O...H	2.970	0.0014	G485:HA2	A484:O
O...H	3.203	0.0083	G485:H	A484:O
O...H	3.498	0.0001	V483:HB	A484:O
N-C	1.353	0.4202	G485:N	A484:C
N...H	3.613	0.0005	G485:N	A484:HB1
N...H	3.953	0.0008	V483:N	A484:H
N...H	3.981	0.0008	G485:N	A484:H
N-N	3.681	0.0053	V483:N	A484:N
C-O	3.644	0.0008	A484:C	C488:O
O...H	2.497	0.0014	A484:HA	C488:O
O...H	3.832	0.0001	A484:HB3	C488:O
H-H	4.063	0.0001	I472:HG23	A484:HA

Table S11: Q493 with their bonding for WT interface model.

Q493				
C-C	3.480	0.0034	Q493:C	S494:C
C-C	3.549	0.0072	Q493:C	S494:CB
C-C	3.835	0.0121	Q493:CA	S494:CA
C-O	3.733	0.0014	Q493:CB	L492:O
C-O	3.964	0.0006	Q493:C	L492:O
C-O	4.087	0.0002	Q493:C	S494:O
C-O	4.434	0.0001	Q493:CG	L492:O
H-C	3.327	0.0004	Q493:H	L492:CB
H-C	3.983	0.0020	Q493:HA	L492:CA
H-C	4.031	0.0007	Q493:HA	S494:CA
H-C	4.380	0.0001	Q493:HG2	L492:C
H-H	4.070	0.0002	Q493:HG3	S494:H
O...H	2.300	0.0074	Q493:HA	L492:O
O...H	3.189	0.0078	Q493:H	L492:O
N-C	1.350	0.4188	Q493:N	L492:C
N...H	3.653	0.0008	Q493:N	L492:HB2
N...H	3.973	0.0009	Q493:N	S494:H
N...H	3.976	0.0006	Q493:N	L492:H
N-N	3.635	0.0052	Q493:N	S494:N
C-C	3.419	0.0021	L492:C	Q493:CB
C-C	3.505	0.0082	L492:C	Q493:C
C-C	3.819	0.0134	L492:CA	Q493:CA
C-O	3.768	0.0002	L492:C	Q493:O
C-O	3.788	0.0008	S494:C	Q493:O
C-O	3.996	0.0012	S494:CB	Q493:O
H-C	3.495	0.0007	S494:HB2	Q493:C
H-C	3.928	0.0001	S494:HB3	Q493:C
H-C	3.985	0.0012	L492:HA	Q493:CA
H-C	4.014	0.0016	S494:HA	Q493:CA
O...H	2.324	0.0062	S494:HA	Q493:O
O...H	3.180	0.0076	S494:H	Q493:O
O...H	3.895	0.0001	S494:HB2	Q493:O
N-C	1.355	0.4486	S494:N	Q493:C
N...H	3.352	0.0002	S494:N	Q493:HB3
N...H	3.825	0.0012	L492:N	Q493:H
N...H	3.969	0.0009	S494:N	Q493:H
N-N	3.540	0.0046	L492:N	Q493:N
C-O	3.577	0.0017	Q493:CD	Y453:OH
C-O	3.676	0.0008	Q493:CD	E35:OE1
C-O	3.895	0.0003	Q493:CD	H34:O
C-O	3.941	0.0007	Q493:CG	Y453:OH
C-O	4.145	0.0011	Q493:CA	Y453:O
C-O	4.240	0.0003	Q493:C	Y453:O
H-C	3.838	0.0006	Q493:HE21	H34:CA
H-C	3.959	0.0001	Q493:H	Y453:CA
H-C	4.000	0.0002	Q493:HB2	Y453:CZ
H-C	4.309	0.0002	Q493:HB2	Y453:CE1
H-C	4.374	0.0001	Q493:HE21	E35:CD
H-C	4.417	0.0001	Q493:HB2	L455:CG
H-C	4.434	0.0001	Q493:H	P491:C
O...H	1.867	0.0533	Q493:HE22	E35:OE1
O...H	2.045	0.0212	Q493:H	Y453:O
O...H	2.165	0.0149	Q493:HE21	H34:O
O...H	3.482	0.0002	Q493:HE22	H34:O
O...H	3.534	0.0005	Q493:HE21	E35:OE1
O...H	3.900	0.0001	Q493:HB2	Y453:OH
O...H	3.964	0.0001	Q493:HG3	Y453:OH
O...H	4.100	0.0010	Q493:HE22	E35:OE2
N-C	3.921	0.0018	Q493:NE2	E35:CD
N-C	4.152	0.0011	Q493:N	Y453:C
N-C	4.259	0.0004	Q493:N	P491:C
N-C	4.305	0.0003	Q493:NE2	E35:CG
N...H	3.790	0.0022	Q493:NE2	Y453:HH
N...H	4.326	0.0001	Q493:N	R454:HA
C-C	4.205	0.0002	H34:C	Q493:CD

Table S12: R493 with their bonding for OV interface model.

R493				
C-C	3.665	0.0121	R493:C	S494:CB
C-C	3.824	0.0127	R493:CA	S494:CA
C-C	4.260	0.0001	R493:CD	S494:CA
C-O	3.520	0.0011	R493:CD	S494:O
C-O	3.660	0.0021	R493:CZ	S494:O
C-O	3.863	0.0003	R493:C	S494:O
C-O	4.135	0.0004	R493:C	L492:O
C-O	4.413	0.0002	R493:CG	L492:O
H-C	3.490	0.0001	R493:HB3	L492:C
H-C	3.971	0.0010	R493:HA	S494:CA
H-C	4.034	0.0013	R493:HA	L492:CA
H-C	4.053	0.0008	R493:HG2	S494:CA
H-C	4.070	0.0002	R493:HB2	L492:CA
H-C	4.438	0.0002	R493:HG2	S494:CB
H-H	3.778	0.0004	R493:HG3	S494:H
H-H	4.305	0.0001	R493:HE	S494:HA
N...H	3.583	0.0008	R493:N	L492:HB2
N...H	3.907	0.0009	R493:N	S494:H
N...H	4.006	0.0005	R493:N	L492:H
N-C	1.349	0.4340	R493:N	L492:C
N-C	3.667	0.0024	R493:NE	S494:C
N-C	4.046	0.0006	R493:NE	S494:CA
N-N	3.626	0.0046	R493:N	S494:N
N-O	3.739	0.0001	R493:NH1	S494:O
O...H	1.699	0.0605	R493:HE	S494:O
O...H	2.391	0.0045	R493:HA	L492:O
O...H	2.652	0.0002	R493:HB2	L492:O
O...H	3.112	0.0023	R493:HD3	S494:O
O...H	3.199	0.0080	R493:H	L492:O
O...H	3.905	0.0001	R493:HB3	L492:O
C-C	3.640	0.0124	L492:C	R493:C
C-C	3.804	0.0140	L492:CA	R493:CA
C-O	3.401	0.0004	S494:C	R493:O
C-O	4.020	0.0005	L492:C	R493:O
C-O	4.215	0.0009	S494:CB	R493:O
H-C	3.186	0.0011	S494:H	R493:CB
H-C	3.707	0.0009	S494:HB2	R493:C
H-C	3.987	0.0011	L492:HA	R493:CA
H-C	3.988	0.0004	S494:HB3	R493:C
H-C	4.036	0.0010	S494:HA	R493:CA
H-C	4.354	0.0001	S494:H	R493:CZ
N...H	3.781	0.0003	S494:N	R493:HB3
N...H	3.885	0.0011	L492:N	R493:H
N...H	3.924	0.0012	S494:N	R493:H
N...H	4.284	0.0003	S494:N	R493:HG3
N...H	4.366	0.0003	S494:N	R493:HD2
N-C	1.357	0.4531	S494:N	R493:C
N-N	3.583	0.0049	L492:N	R493:N
O...H	2.526	0.0030	S494:HA	R493:O
O...H	3.163	0.0086	S494:H	R493:O
C-O	3.861	0.0018	R493:CZ	E35:OE1
C-O	4.078	0.0013	R493:CA	Y453:O
C-O	4.155	0.0003	R493:C	Y453:O
C-O	4.207	0.0002	R493:CD	E35:OE1
H-C	3.287	0.0027	R493:HH22	H34:CA
H-C	3.635	0.0012	R493:HH21	H34:C
H-C	3.759	0.0004	R493:HH22	E35:CB
H-C	4.184	0.0002	R493:HH12	D38:CA
H-C	4.197	0.0001	R493:HH12	H34:CA
H-C	4.246	0.0001	R493:HE	Y453:CE1
H-C	4.412	0.0002	R493:HH12	D38:CG
N...H	4.084	0.0001	R493:NH1	D38:HB3
N...H	4.271	0.0001	R493:NH2	D38:HB2
N...H	4.424	0.0001	R493:N	R454:HA
N-C	3.707	0.0016	R493:NH2	E35:CD

Table S11: Q493 with their bonding for WT interface model.

Q493				
C-C	4.306	0.0007	Y453:CZ	Q493:CD
C-C	4.309	0.0004	Y453:CE1	Q493:CD
C-C	4.483	0.0005	Y453:CZ	Q493:CG
C-C	4.485	0.0008	L452:CA	Q493:C
C-O	3.372	0.0017	Y453:CZ	Q493:OE1
C-O	3.697	0.0010	L452:C	Q493:O
C-O	4.085	0.0012	Y453:CA	Q493:O
C-O	4.173	0.0002	Y453:C	Q493:O
C-O	4.419	0.0002	L452:CG	Q493:O
C-O	4.450	0.0001	H34:C	Q493:OE1
H-C	3.686	0.0002	Y453:HE1	Q493:CD
H-C	4.002	0.0001	Y453:H	Q493:CA
H-C	4.026	0.0001	H34:HB3	Q493:CG
H-C	4.054	0.0002	L452:HD21	Q493:C
H-C	4.160	0.0001	Y453:HE1	Q493:CA
H-C	4.231	0.0001	Y495:H	Q493:C
H-C	4.243	0.0002	Y453:HE1	Q493:CG
H-C	4.413	0.0002	Y453:HH	Q493:CA
H-H	3.919	0.0001	H34:HB3	Q493:HE22
H-H	3.977	0.0001	Y453:HH	Q493:HE21
H-H	4.222	0.0001	H34:HA	Q493:HE21
H-H	4.248	0.0001	L455:HB2	Q493:HG3
H-H	4.294	0.0001	L455:HD22	Q493:HG3
H-H	4.297	0.0001	Y453:HH	Q493:HG2
H-H	4.462	0.0001	Y453:HE1	Q493:HB2
O...H	1.643	0.0887	Y453:HH	Q493:OE1
O...H	2.022	0.0220	Y453:H	Q493:O
O...H	2.453	0.0029	L452:HA	Q493:O
O...H	3.256	0.0001	L452:HB3	Q493:O
N-C	4.205	0.0003	Y495:N	Q493:C
N-C	4.211	0.0016	Y453:N	Q493:C
N...H	4.138	0.0003	R454:N	Q493:H
N-N	3.822	0.0003	E35:N	Q493:NE2

Table S12: R493 with their bonding for OV interface model.

R493				
N-C	4.004	0.0001	R493:NH2	E35:CG
N-C	4.021	0.0017	R493:NH1	H34:C
N-C	4.140	0.0017	R493:N	Y453:C
N-C	4.288	0.0001	R493:NH2	E35:CB
N-C	4.327	0.0003	R493:N	P491:C
N-C	4.498	0.0001	R493:NE	Y453:CE1
N-O	4.273	0.0004	R493:NE	E35:OE1
N-O	4.423	0.0001	R493:NE	Y453:OH
O...H	1.804	0.0616	R493:HH21	E35:OE1
O...H	1.809	0.0407	R493:HH22	H34:O
O...H	1.979	0.0253	R493:H	Y453:O
O...H	2.136	0.0135	R493:HH12	H34:O
O...H	3.176	0.0001	R493:HH22	E35:OE1
O...H	3.548	0.0010	R493:HH21	H34:O
O...H	3.713	0.0004	R493:HH11	H34:O
O...H	4.034	0.0012	R493:HH21	E35:OE2
O...H	4.277	0.0001	R493:HD2	E35:OE1
O...H	4.349	0.0001	R493:HH22	E35:O
C-C	3.972	0.0018	H34:C	R493:CZ
C-O	3.669	0.0012	L452:C	R493:O
C-O	3.909	0.0018	Y453:CA	R493:O
C-O	4.025	0.0003	Y453:C	R493:O
H-C	3.841	0.0007	E35:HA	R493:CZ
H-C	3.890	0.0002	L452:HD21	R493:C
H-C	3.908	0.0007	D38:HB2	R493:CZ
H-C	3.964	0.0002	Y453:H	R493:CA
H-C	4.214	0.0001	Y495:H	R493:C
H-H	3.092	0.0003	E35:HA	R493:HH21
H-H	3.297	0.0001	D38:HB2	R493:HH11
H-H	3.483	0.0001	H34:HB3	R493:HH21
H-H	3.592	0.0001	D38:HB3	R493:HH12
H-H	3.685	0.0001	Y453:HE1	R493:HD2
H-H	3.788	0.0003	H34:HA	R493:HH22
N...H	3.892	0.0017	Y495:N	R493:HE
N...H	4.137	0.0002	E35:N	R493:HH12
N...H	4.176	0.0004	R454:N	R493:H
N-C	4.121	0.0026	Y453:N	R493:C
N-C	4.144	0.0003	Y495:N	R493:C
N-N	3.421	0.0019	E35:N	R493:NH2
N-N	4.432	0.0003	Y453:N	R493:N
O...H	1.895	0.0288	Y453:H	R493:O
O...H	2.548	0.0018	L452:HA	R493:O
O...H	3.521	0.0001	L452:HB3	R493:O
O...H	3.754	0.0001	Y495:H	R493:O

Table S13: G496 with their bonding for WT interface model.

G496				
C-C	3.687	0.0117	G496:C	F497:CB
C-C	3.813	0.0144	G496:CA	F497:CA
C-O	4.030	0.0001	G496:C	F497:O
H-C	3.339	0.0116	G496:HA3	Y495:C
H-C	3.851	0.0012	G496:H	Y495:CB
H-C	3.975	0.0010	G496:HA2	F497:CA
H-C	4.167	0.0005	G496:HA2	Y495:CA
H-H	3.482	0.0011	G496:HA3	F497:H
O...H	2.604	0.0043	G496:HA2	Y495:O
O...H	3.142	0.0078	G496:H	Y495:O
O...H	3.829	0.0003	G496:HA3	Y495:O
N-C	1.360	0.4246	G496:N	Y495:C
N-C	3.543	0.0037	G496:N	Y495:CB
N...H	3.391	0.0006	G496:N	Y495:HB3
N...H	4.035	0.0004	G496:N	Y495:HB2
C-C	3.822	0.0142	Y495:CA	G496:CA
C-O	3.618	0.0008	F497:C	G496:O
C-O	4.051	0.0002	Y495:C	G496:O
C-O	4.211	0.0006	F497:CB	G496:O
H-C	3.918	0.0012	Y495:HA	G496:CA
H-C	4.004	0.0002	F497:HD1	G496:C
H-C	4.021	0.0004	F497:HB2	G496:C
H-C	4.068	0.0009	F497:HA	G496:CA
H-H	3.847	0.0001	Y495:HB3	G496:H
O...H	2.472	0.0041	F497:HA	G496:O
O...H	3.157	0.0077	F497:H	G496:O
N-C	1.350	0.4694	F497:N	G496:C
N...H	3.288	0.0047	F497:N	G496:HA3
N...H	3.307	0.0008	Y495:N	G496:H
N-N	3.370	0.0033	Y495:N	G496:N
C-O	4.142	0.0001	G496:C	D38:OD1
C-O	4.326	0.0008	G496:CA	S494:O
H-C	4.254	0.0004	G496:HA3	D38:CB
H-C	4.360	0.0006	G496:HA3	K353:CE
O...H	2.322	0.0066	G496:HA3	D38:OD1
O...H	2.494	0.0064	G496:H	S494:O
O...H	3.901	0.0001	G496:HA2	D38:OD1
O...H	3.979	0.0003	G496:H	D38:OD1
O...H	4.497	0.0001	G496:HA3	S494:O
N-C	4.434	0.0001	G496:N	Y449:CE1
N...H	3.573	0.0006	G496:N	K353:HZ1
N...H	3.956	0.0002	G496:N	K353:HZ3
N-O	4.187	0.0002	G496:N	D38:OD1
C-O	3.886	0.0019	K353:CE	G496:O
C-O	4.259	0.0005	N501:CG	G496:O
H-C	3.724	0.0005	K353:HZ3	G496:C
H-C	3.815	0.0002	Q498:H	G496:C
H-C	4.395	0.0001	N448:H	G496:C
H-H	3.710	0.0003	K353:HZ3	G496:H
H-H	4.036	0.0003	K353:HZ1	G496:HA2
H-H	4.233	0.0001	K353:HZ3	G496:HA2
O...H	1.793	0.1000	K353:HZ1	G496:O
O...H	2.280	0.0126	N501:HD21	G496:O
O...H	3.725	0.0003	N501:HD22	G496:O
N-C	3.535	0.0009	K353:NZ	G496:C
N-C	3.905	0.0004	Q498:N	G496:C
N-C	4.264	0.0006	N501:ND2	G496:C

Table S14: S496 with their bonding for OV interface model.

S496				
C-C	3.471	0.0001	S496:C	F497:C
C-C	3.656	0.0107	S496:C	F497:CB
C-C	3.820	0.0136	S496:CA	F497:CA
C-O	3.351	0.0001	S496:C	Y495:O
C-O	4.000	0.0002	S496:C	F497:O
C-O	4.236	0.0007	S496:CB	Y495:O
H-C	3.764	0.0008	S496:HB2	Y495:C
H-C	3.929	0.0012	S496:HA	F497:CA
H-C	3.931	0.0014	S496:H	Y495:CB
H-C	3.984	0.0005	S496:HB3	Y495:C
H-C	4.065	0.0010	S496:HA	Y495:CA
N...H	3.122	0.0002	S496:N	F497:H
N...H	3.671	0.0015	S496:N	Y495:HB3
N...H	3.786	0.0001	S496:N	Y495:H
N...H	4.298	0.0001	S496:N	Y495:HD1
N-C	1.362	0.4151	S496:N	Y495:C
N-C	3.699	0.0057	S496:N	Y495:CB
N-N	3.234	0.0003	S496:N	F497:N
O...H	2.561	0.0045	S496:HA	Y495:O
O...H	3.165	0.0085	S496:H	Y495:O
C-C	3.675	0.0129	Y495:C	S496:CB
C-C	3.813	0.0138	Y495:CA	S496:CA
C-O	3.761	0.0006	F497:C	S496:O
C-O	4.084	0.0001	Y495:C	S496:O
C-O	4.205	0.0006	F497:CB	S496:O
H-C	3.852	0.0017	F497:H	S496:CB
H-C	3.958	0.0012	Y495:HA	S496:CA
H-C	4.044	0.0002	F497:HB2	S496:C
H-C	4.049	0.0011	F497:HA	S496:CA
H-C	4.146	0.0001	F497:HD1	S496:C
H-H	4.098	0.0001	Y495:HD1	S496:H
N...H	4.140	0.0004	F497:N	S496:HB3
N-C	1.358	0.4463	F497:N	S496:C
N-C	3.656	0.0052	F497:N	S496:CB
O...H	2.477	0.0053	F497:HA	S496:O
O...H	3.160	0.0080	F497:H	S496:O
C-C	4.255	0.0014	S496:C	Y501:CE2
C-O	4.123	0.0013	S496:CA	S494:O
C-O	4.174	0.0001	S496:CB	D38:OD2
C-O	4.498	0.0001	S496:CB	Y449:OH
H-C	3.855	0.0003	S496:HB2	Y449:CZ
H-C	3.861	0.0033	S496:HG	D38:CB
H-C	4.073	0.0009	S496:H	S494:CA
H-C	4.090	0.0002	S496:HA	Y449:CD1
H-C	4.265	0.0001	S496:HA	Y449:CG
H-C	4.283	0.0001	S496:HG	Y449:CD1
H-C	4.329	0.0002	S496:HB3	Y449:CE1
H-C	4.436	0.0001	S496:HA	Y449:CE1
H-C	4.474	0.0002	S496:HB3	K353:CE
H-H	3.247	0.0005	S496:HG	R498:HH11
H-H	3.881	0.0002	S496:HB3	R498:HH12
N...H	4.218	0.0001	S496:N	Y449:HA
N...H	4.287	0.0001	S496:N	K353:HZ2
O...H	1.751	0.0658	S496:HG	D38:OD1
O...H	2.203	0.0090	S496:H	S494:O
O...H	2.833	0.0016	S496:HB3	D38:OD1
C-O	3.935	0.0003	Y501:CZ	S496:O
C-O	4.217	0.0012	K353:CE	S496:O
C-O	4.305	0.0006	Y501:CD2	S496:O
C-O	4.360	0.0001	Y449:CG	S496:OG
C-O	4.406	0.0001	Y449:CD2	S496:OG
H-C	3.919	0.0011	R498:HH12	S496:CB
H-C	4.150	0.0002	Y449:HH	S496:CB
H-C	4.166	0.0001	K353:HZ1	S496:C
H-C	4.223	0.0001	Y449:HA	S496:CB
H-C	4.264	0.0001	K353:HZ3	S496:C
H-C	4.359	0.0001	R498:H	S496:C
H-C	4.414	0.0001	N448:H	S496:C
H-H	3.436	0.0001	K353:HZ2	S496:HG
H-H	3.772	0.0001	Y449:HA	S496:HB2
H-H	3.918	0.0003	K353:HZ1	S496:HB3
H-H	3.981	0.0001	Y449:HE1	S496:HB3
N...H	4.496	0.0001	R498:NH1	S496:HB3
N-C	4.095	0.0002	K353:NZ	S496:CB
N-C	4.097	0.0013	K353:NZ	S496:C
N-C	4.306	0.0002	R498:N	S496:C
N-C	4.432	0.0006	R498:NH1	S496:CB
O...H	2.108	0.0392	K353:HZ2	S496:O
O...H	2.213	0.0016	Y501:HE2	S496:O
O...H	4.358	0.0002	Y501:HD2	S496:O
O...H	4.426	0.0001	K353:HE2	S496:O

Table S15: Q498 with their bonding for WT interface model.

Q498				
C-C	3.662	0.0134	Q498:C	P499:CB
C-C	3.676	0.0120	Q498:C	P499:CG
C-C	3.870	0.0120	Q498:CA	P499:CA
C-C	4.446	0.0006	Q498:CA	P499:CG
C-C	4.457	0.0001	Q498:CG	P499:CD
C-O	4.022	0.0007	Q498:CB	F497:O
C-O	4.027	0.0005	Q498:C	F497:O
C-O	4.235	0.0001	Q498:C	P499:O
H-C	3.608	0.0018	Q498:H	F497:CB
H-C	3.795	0.0002	Q498:HB2	F497:C
H-C	4.056	0.0002	Q498:HG3	F497:C
H-C	4.094	0.0012	Q498:HA	F497:CA
H-C	4.100	0.0005	Q498:HA	P499:CA
H-C	4.180	0.0008	Q498:HA	P499:CG
H-C	4.268	0.0002	Q498:HB3	P499:CG
H-C	4.424	0.0002	Q498:HB3	P499:C
H-H	3.899	0.0001	Q498:HB3	P499:HD3
H-H	3.941	0.0001	Q498:HG2	P499:HD2
H-H	4.024	0.0001	Q498:HB2	P499:HD2
H-H	4.095	0.0001	Q498:HB3	P499:HG2
H-H	4.181	0.0001	Q498:HG3	P499:HD3
H-H	4.385	0.0001	Q498:HA	P499:HA
N...H	3.987	0.0004	Q498:N	F497:HB2
N...H	4.233	0.0002	Q498:N	P499:HD3
N...H	4.240	0.0002	Q498:N	P499:HD2
N...H	4.374	0.0001	Q498:N	F497:HD1
N-C	1.338	0.4593	Q498:N	F497:C
N-C	3.475	0.0024	Q498:N	F497:CB
N-C	4.271	0.0011	Q498:N	P499:CD
N-N	3.584	0.0045	Q498:N	P499:N
O...H	2.497	0.0046	Q498:HA	F497:O
O...H	3.190	0.0083	Q498:H	F497:O
C-C	3.496	0.0066	F497:C	Q498:C
C-C	3.576	0.0054	F497:C	Q498:CB
C-C	3.825	0.0144	F497:CA	Q498:CA
C-C	4.028	0.0006	F497:C	Q498:CG
C-O	3.658	0.0097	P499:CD	Q498:O
C-O	3.820	0.0006	F497:C	Q498:O
C-O	4.227	0.0011	P499:CB	Q498:O
H-C	3.877	0.0015	F497:HA	Q498:CA
H-C	4.023	0.0003	P499:HG2	Q498:C
H-C	4.055	0.0002	P499:HB3	Q498:C
H-C	4.124	0.0006	P499:HA	Q498:CA
H-H	3.450	0.0002	F497:HB3	Q498:H
H-H	3.792	0.0001	F497:HD1	Q498:H
N...H	3.511	0.0017	F497:N	Q498:H
N...H	3.888	0.0002	P499:N	Q498:HB2
N...H	3.911	0.0011	P499:N	Q498:H
N-C	1.358	0.4974	P499:N	Q498:C
N-N	3.393	0.0026	F497:N	Q498:N
O...H	2.669	0.0009	P499:HA	Q498:O
O...H	3.981	0.0001	F497:H	Q498:OE1
O...H	4.009	0.0005	P499:HD2	Q498:O
O...H	4.082	0.0003	P499:HD3	Q498:O
C-C	4.318	0.0004	Q498:CA	N501:CG
C-C	4.403	0.0004	Q498:C	T500:CA
C-O	3.989	0.0002	Q498:CA	N501:OD1
C-O	4.184	0.0002	Q498:C	S443:OG
C-O	4.342	0.0006	Q498:CD	Q42:OE1
C-O	4.419	0.0001	Q498:CB	K444:O
H-C	3.782	0.0006	Q498:HE22	Q42:CG
H-C	3.815	0.0002	Q498:H	G496:C
H-C	4.017	0.0001	Q498:HG2	Y41:CD2
H-C	4.061	0.0001	Q498:HG3	G446:CA
H-C	4.151	0.0004	Q498:HB3	T500:CB

Table S16: R498 with their bonding for OV interface model.

R498				
C-C	3.664	0.0131	R498:C	P499:CB
C-C	3.684	0.0121	R498:C	P499:CG
C-C	3.877	0.0121	R498:CA	P499:CA
C-C	4.393	0.0003	R498:CG	P499:CD
C-C	4.423	0.0006	R498:CA	P499:CG
C-O	3.903	0.0005	R498:CB	F497:O
C-O	4.017	0.0002	R498:CD	F497:O
C-O	4.099	0.0005	R498:C	F497:O
H-C	3.383	0.0009	R498:H	F497:CB
H-C	3.888	0.0001	R498:HB2	F497:C
H-C	3.931	0.0004	R498:HB3	P499:CG
H-C	4.068	0.0012	R498:HA	F497:CA
H-C	4.140	0.0004	R498:HA	P499:CA
H-C	4.204	0.0004	R498:HB2	P499:CD
H-C	4.224	0.0007	R498:HA	P499:CG
H-C	4.437	0.0001	R498:HB3	P499:C
H-H	3.552	0.0005	R498:HB3	P499:HD3
H-H	3.668	0.0002	R498:HG2	P499:HD2
H-H	3.690	0.0004	R498:HB2	P499:HD2
H-H	3.785	0.0002	R498:HB3	P499:HG2
H-H	4.407	0.0001	R498:HA	P499:HA
N...H	3.738	0.0009	R498:N	F497:HB2
N...H	4.257	0.0002	R498:N	P499:HD2
N...H	4.320	0.0002	R498:N	P499:HD3
N-C	1.347	0.4587	R498:N	F497:C
N-C	4.325	0.0011	R498:N	P499:CD
N-N	3.646	0.0042	R498:N	P499:N
O...H	2.485	0.0050	R498:HA	F497:O
O...H	3.192	0.0082	R498:H	F497:O
C-C	3.530	0.0068	F497:C	R498:C
C-C	3.573	0.0057	F497:C	R498:CB
C-C	3.839	0.0002	F497:C	R498:CG
C-C	3.842	0.0130	F497:CA	R498:CA
C-O	3.665	0.0094	P499:CD	R498:O
C-O	3.768	0.0007	F497:C	R498:O
C-O	4.253	0.0009	P499:CB	R498:O
H-C	3.974	0.0011	F497:HA	R498:CA
H-C	4.040	0.0003	P499:HB3	R498:C
H-C	4.059	0.0002	P499:HG2	R498:C
H-C	4.140	0.0006	P499:HA	R498:CA
H-H	3.937	0.0001	F497:HD1	R498:H
N...H	3.632	0.0002	P499:N	R498:HB2
N...H	3.801	0.0012	F497:N	R498:H
N...H	3.823	0.0019	P499:N	R498:H
N-C	1.358	0.4864	P499:N	R498:C
N-N	3.588	0.0041	F497:N	R498:N
O...H	2.665	0.0008	P499:HA	R498:O
O...H	4.043	0.0005	P499:HD3	R498:O
O...H	4.045	0.0003	P499:HD2	R498:O
C-C	4.455	0.0001	R498:CG	Y501:CZ
C-C	4.464	0.0002	R498:CA	Y501:CZ
C-C	4.481	0.0005	R498:CA	Y501:CE2
C-O	3.628	0.0007	R498:CZ	S446:O
C-O	3.858	0.0014	R498:CZ	Y449:OH
C-O	3.902	0.0014	R498:CZ	D38:OD1
C-O	3.905	0.0014	R498:CZ	Y501:OH
C-O	4.101	0.0002	R498:C	S443:OG
C-O	4.262	0.0001	R498:CB	K444:O
C-O	4.359	0.0007	R498:CG	Y501:OH
C-O	4.426	0.0002	R498:CB	Y501:OH
C-O	4.426	0.0002	R498:C	N439:OD1
H-C	3.733	0.0003	R498:HH21	Y449:CZ
H-C	3.855	0.0014	R498:HH22	Y449:CE1
H-C	3.919	0.0011	R498:HH12	S496:CB
H-C	3.955	0.0002	R498:HH22	D38:CG
H-C	3.983	0.0001	R498:HH21	Q42:CG
H-C	3.986	0.0016	R498:HH21	S446:CA
H-C	4.116	0.0005	R498:HH11	Y501:CE1
H-C	4.123	0.0013	R498:HH12	D38:CB
H-C	4.175	0.0001	R498:HD3	Y41:CZ
H-C	4.213	0.0001	R498:HD3	Y41:CG
H-C	4.235	0.0001	R498:HH11	Y41:CG
H-C	4.259	0.0001	R498:HB3	T500:CB
H-C	4.265	0.0001	R498:HH12	Y449:CZ
H-C	4.272	0.0003	R498:HH22	Y449:CD2
H-C	4.289	0.0001	R498:HA	K444:CA

Table S15: Q498 with their bonding for WT interface model.

Q498				
H-C	4.219	0.0001	Q498:HG3	V445:C
H-C	4.377	0.0001	Q498:HB3	T500:CA
H-C	4.405	0.0001	Q498:HA	K444:CA
H-C	4.458	0.0002	Q498:HB2	N501:CB
H-H	3.210	0.0005	Q498:H	N501:HD22
H-H	3.678	0.0001	Q498:HB2	T500:H
N...H	3.704	0.0001	Q498:NE2	Y41:HE2
N...H	4.107	0.0008	Q498:N	N448:H
N-C	3.905	0.0004	Q498:N	G496:C
O...H	2.276	0.0082	Q498:HE22	Q42:OE1
O...H	2.429	0.0044	Q498:HB2	N501:OD1
O...H	2.857	0.0005	Q498:HA	K444:O
O...H	3.027	0.0001	Q498:HG3	K444:O
O...H	3.828	0.0001	Q498:HB3	N501:OD1
C-O	3.938	0.0007	N501:CA	Q498:O
C-O	4.160	0.0007	T500:C	Q498:O
C-O	4.195	0.0005	T500:CA	Q498:O
C-O	4.478	0.0003	G447:C	Q498:OE1
H-C	3.969	0.0002	G447:H	Q498:CD
H-C	4.075	0.0006	N501:H	Q498:CA
H-C	4.159	0.0001	Y41:HD2	Q498:CG
H-C	4.215	0.0001	N501:HB2	Q498:C
H-C	4.243	0.0001	G447:HA2	Q498:CB
H-C	4.361	0.0002	N501:HD21	Q498:CA
H-C	4.444	0.0001	K444:H	Q498:C
H-C	4.450	0.0001	G446:H	Q498:CD
H-C	4.476	0.0001	G447:HA3	Q498:CG
H-H	3.417	0.0001	Y41:HE2	Q498:HE22
H-H	3.654	0.0002	Y41:HD2	Q498:HG2
H-H	4.140	0.0002	Y41:HE2	Q498:HG3
H-H	4.259	0.0001	G447:H	Q498:HG2
N...H	4.405	0.0001	G447:N	Q498:HG2
N...H	4.444	0.0001	N501:N	Q498:HB3
N-C	3.765	0.0007	G447:N	Q498:CD
N-C	4.397	0.0002	T500:N	Q498:CB
N-C	4.436	0.0005	N501:ND2	Q498:CA
N-C	4.479	0.0001	N501:ND2	Q498:C
N-O	3.996	0.0003	G447:N	Q498:OE1
O...H	2.179	0.0169	N501:H	Q498:O
O...H	2.381	0.0023	G447:HA2	Q498:OE1
O...H	4.136	0.0001	N501:HD22	Q498:O
O...H	4.241	0.0001	G447:H	Q498:OE1

Table S16: R498 with their bonding for OV interface model.

R498				
H-C	4.309	0.0001	R498:HG3	S446:CA
H-C	4.312	0.0001	R498:HA	S443:CA
H-C	4.359	0.0001	R498:H	S496:C
H-C	4.371	0.0001	R498:HH22	G447:CA
H-C	4.406	0.0002	R498:HB3	Y501:CE1
H-C	4.424	0.0002	R498:HG3	G447:C
H-C	4.428	0.0002	R498:HA	S443:C
H-C	4.430	0.0001	R498:HH21	Y449:CD2
H-C	4.431	0.0003	R498:HE	G447:C
H-C	4.432	0.0003	R498:HH11	K353:CE
H-C	4.436	0.0002	R498:HH11	D38:CG
H-C	4.467	0.0001	R498:HH11	Y41:CE2
H-C	4.497	0.0002	R498:HH22	S446:C
H-H	3.297	0.0005	R498:HD2	Y501:HH
H-H	3.660	0.0004	R498:HH12	Y501:HH
H-H	3.833	0.0001	R498:HB2	Y501:HH
N...H	4.029	0.0001	R498:NH1	K353:HZ2
N...H	4.100	0.0002	R498:NE	Y501:HH
N...H	4.195	0.0007	R498:N	N448:H
N...H	4.483	0.0001	R498:NE	Y41:HE2
N...H	4.496	0.0001	R498:NH1	S496:HB3
N-C	3.695	0.0004	R498:NH2	S446:C
N-C	3.904	0.0014	R498:NH1	D38:CG
N-C	4.211	0.0014	R498:NH1	Y501:CZ
N-C	4.306	0.0002	R498:N	S496:C
N-C	4.332	0.0002	R498:N	Y501:CE1
N-C	4.432	0.0006	R498:NH1	S496:CB
N-C	4.440	0.0001	R498:N	S443:CB
N-O	3.957	0.0008	R498:NE	Y501:OH
N-O	4.056	0.0003	R498:NH1	Y449:OH
N-O	4.143	0.0002	R498:NH2	D38:OD1
N-O	4.337	0.0001	R498:NH1	D38:OD2
N-O	4.410	0.0001	R498:N	Y501:OH
O...H	1.863	0.0263	R498:HH22	Y449:OH
O...H	1.867	0.0525	R498:HH21	S446:O
O...H	1.886	0.0345	R498:HH12	D38:OD1
O...H	1.948	0.0086	R498:HH11	Y501:OH
O...H	2.799	0.0004	R498:HG3	K444:O
O...H	2.988	0.0001	R498:HA	K444:O
O...H	3.254	0.0003	R498:HH11	D38:OD1
O...H	3.316	0.0005	R498:HH21	Y449:OH
O...H	3.434	0.0004	R498:HH12	Y501:OH
O...H	3.537	0.0008	R498:HH22	S446:O
O...H	4.303	0.0001	R498:HA	S443:OG
C-C	4.080	0.0010	S446:C	R498:CZ
C-C	4.449	0.0002	Y449:CZ	R498:CZ
C-O	4.442	0.0003	T500:CA	R498:O
C-O	4.487	0.0001	Y501:CA	R498:O
H-C	4.066	0.0005	Y501:HH	R498:CZ
H-C	4.071	0.0002	G447:HA3	R498:CD
H-C	4.140	0.0003	Y449:HH	R498:CZ
H-C	4.185	0.0006	Y449:HE2	R498:CZ
H-C	4.304	0.0002	G447:HA2	R498:CB
H-C	4.348	0.0002	G447:H	R498:CD
H-C	4.356	0.0001	Q42:HG3	R498:CZ
H-C	4.357	0.0004	K353:HZ1	R498:CZ
H-C	4.429	0.0001	Y501:HE1	R498:CG
H-C	4.448	0.0001	K353:HZ1	R498:CD
H-C	4.461	0.0001	K444:H	R498:C
H-C	4.482	0.0001	Q42:HE21	R498:CZ
H-C	4.483	0.0001	G447:HA3	R498:CG
H-C	4.493	0.0001	S443:HB3	R498:CA
H-H	3.218	0.0002	Y449:HH	R498:HH12
H-H	3.247	0.0005	S496:HG	R498:HH11
H-H	3.359	0.0003	G447:HA3	R498:HH22
H-H	3.531	0.0002	K353:HZ2	R498:HH11
H-H	3.645	0.0005	G447:HA2	R498:HH21
H-H	3.750	0.0002	K353:HZ2	R498:HH12
H-H	3.871	0.0001	K353:HZ1	R498:HD2
H-H	3.872	0.0001	G447:H	R498:HG2
H-H	3.881	0.0002	S496:HB3	R498:HH12
H-H	3.888	0.0001	Q42:HG2	R498:HH21
H-H	3.900	0.0001	Q42:HE21	R498:HH22
H-H	3.993	0.0001	Q42:HG2	R498:HH11
H-H	4.050	0.0002	Y449:HH	R498:HH21
H-H	4.081	0.0002	K353:HZ1	R498:HD3

Table S16: R498 with their bonding for OV interface model.

R498				
H-H	4.087	0.0001	G447:HA2	R498:HG2
H-H	4.112	0.0001	K353:HD2	R498:HH11
H-H	4.119	0.0001	S443:HA	R498:HA
H-H	4.134	0.0001	L45:HD23	R498:HD3
H-H	4.229	0.0001	S443:HB3	R498:HA
N-C	4.157	0.0006	G447:N	R498:CZ
N-C	4.158	0.0005	G447:N	R498:CD
N-C	4.299	0.0002	Q42:NE2	R498:CZ
N-C	4.349	0.0001	T500:N	R498:CB
N-N	3.699	0.0001	K353:NZ	R498:NH1
N-N	4.057	0.0014	G447:N	R498:NH2
O...H	2.854	0.0016	Y501:H	R498:O

Table S17: N501 with their bonding for WT interface model.

N501				
C-C	3.795	0.0143	N501:CA	G502:CA
C-C	4.379	0.0003	N501:CA	G502:C
C-O	2.915	0.0007	N501:C	T500:O
C-O	4.280	0.0006	N501:CB	T500:O
H-C	3.713	0.0011	N501:H	T500:CB
H-C	3.962	0.0008	N501:HB2	T500:C
H-C	4.004	0.0009	N501:HA	G502:CA
H-C	4.180	0.0003	N501:HA	T500:CA
N...H	3.172	0.0014	N501:N	T500:HA
N...H	3.734	0.0012	N501:N	T500:HB
N...H	3.955	0.0009	N501:N	G502:H
N-C	1.352	0.4225	N501:N	T500:C
N-C	3.525	0.0010	N501:N	T500:CB
N-N	3.625	0.0049	N501:N	G502:N
O...H	2.763	0.0017	N501:HA	T500:O
O...H	3.157	0.0002	N501:HB3	G502:O
O...H	3.189	0.0088	N501:H	T500:O
C-C	3.734	0.0147	T500:C	N501:CB
C-C	3.831	0.0137	T500:CA	N501:CA
C-C	4.367	0.0004	T500:C	N501:CG
C-C	4.393	0.0005	T500:CA	N501:C
H-C	3.302	0.0120	G502:HA3	N501:C
H-C	4.157	0.0005	G502:HA2	N501:CA
H-C	4.479	0.0001	T500:H	N501:CA
H-H	3.399	0.0008	T500:HA	N501:H
N...H	3.746	0.0007	G502:N	N501:HB2
N-C	1.352	0.4478	G502:N	N501:C
N-C	4.216	0.0010	T500:N	N501:CA
O...H	2.586	0.0008	G502:HA2	N501:O
O...H	3.198	0.0081	G502:H	N501:O
O...H	3.773	0.0006	G502:HA3	N501:O
C-O	3.842	0.0007	N501:CG	Y505:O
C-O	3.851	0.0015	N501:C	K353:O
C-O	3.938	0.0007	N501:CA	Q498:O
C-O	4.259	0.0005	N501:CG	G496:O
H-C	3.891	0.0004	N501:HD22	Y505:CA
H-C	3.896	0.0004	N501:HD21	F497:C
H-C	4.075	0.0006	N501:H	Q498:CA
H-C	4.100	0.0003	N501:HB2	Q506:CB
H-C	4.215	0.0001	N501:HB2	Q498:C
H-C	4.294	0.0002	N501:HD21	K353:CE
H-C	4.361	0.0002	N501:HD21	Q498:CA
H-C	4.420	0.0002	N501:HB2	Q506:CD
H-H	3.725	0.0002	N501:HD22	Y505:HB2
H-H	3.739	0.0001	N501:HD21	Y505:HB3
H-H	3.824	0.0001	N501:HB3	Q506:HA
H-H	4.060	0.0001	N501:HB3	Q506:HG2
H-H	4.200	0.0001	N501:HB2	Y505:HB2
N...H	4.265	0.0001	N501:ND2	Y505:HB2
N...H	4.444	0.0001	N501:N	Q498:HB3
N-C	3.951	0.0008	N501:ND2	Y505:C
N-C	4.264	0.0006	N501:ND2	G496:C
N-C	4.264	0.0006	N501:ND2	F497:C
N-C	4.436	0.0005	N501:ND2	Q498:CA
N-C	4.479	0.0001	N501:ND2	Q498:C
O...H	1.942	0.0359	N501:HD22	Y505:O
O...H	2.179	0.0169	N501:H	Q498:O
O...H	2.280	0.0126	N501:HD21	G496:O
O...H	3.562	0.0005	N501:HD21	Y505:O
O...H	3.725	0.0003	N501:HD22	G496:O
O...H	4.136	0.0001	N501:HD22	Q498:O

Table S18: Y501 with their bonding for OV interface model.

Y501				
C-C	3.760	0.0149	Y501:CA	G502:CA
C-C	4.257	0.0007	Y501:CA	G502:C
C-C	4.422	0.0001	Y501:CB	G502:C
C-O	3.339	0.0003	Y501:C	T500:O
C-O	4.232	0.0007	Y501:CB	T500:O
H-C	3.837	0.0010	Y501:H	T500:CB
H-C	3.941	0.0010	Y501:HA	G502:CA
H-C	3.973	0.0007	Y501:HB2	T500:C
H-C	4.051	0.0011	Y501:HA	T500:CA
H-H	3.898	0.0001	Y501:HB2	G502:H
N...H	3.799	0.0014	Y501:N	T500:HB
N...H	3.972	0.0009	Y501:N	G502:H
N-C	1.357	0.4130	Y501:N	T500:C
N-C	3.621	0.0023	Y501:N	T500:CB
N-N	3.666	0.0050	Y501:N	G502:N
N-O	3.806	0.0001	Y501:N	T500:OG1
O...H	2.525	0.0055	Y501:HA	T500:O
O...H	3.039	0.0004	Y501:HB3	G502:O
O...H	3.172	0.0085	Y501:H	T500:O
C-C	3.682	0.0144	T500:C	Y501:CB
C-C	3.841	0.0132	T500:CA	Y501:CA
C-C	4.106	0.0004	T500:C	Y501:CG
H-C	3.297	0.0124	G502:HA3	Y501:C
H-C	4.194	0.0003	G502:HA2	Y501:CA
H-H	3.247	0.0008	T500:HA	Y501:H
H-H	3.922	0.0001	T500:HB	Y501:HD1
N...H	3.711	0.0009	G502:N	Y501:HB2
N...H	4.415	0.0001	G502:N	Y501:HD1
N-C	1.352	0.4468	G502:N	Y501:C
N-C	4.212	0.0008	T500:N	Y501:CA
N-C	4.346	0.0001	G502:N	Y501:CG
O...H	3.203	0.0079	G502:H	Y501:O
O...H	3.779	0.0007	G502:HA3	Y501:O
C-O	3.697	0.0008	Y501:CE1	Y41:OH
C-O	3.935	0.0003	Y501:CZ	S496:O
C-O	3.937	0.0012	Y501:C	K353:O
C-O	4.305	0.0006	Y501:CD2	S496:O
C-O	4.396	0.0002	Y501:CG	H505:O
C-O	4.478	0.0002	Y501:CE2	H505:O
C-O	4.487	0.0001	Y501:CA	R498:O
H-C	2.359	0.0208	Y501:HH	Y41:CD2
H-C	2.494	0.0140	Y501:HH	Y41:CG
H-C	3.560	0.0007	Y501:HD1	Y41:CZ
H-C	3.657	0.0002	Y501:HD1	Y41:CE1
H-C	3.783	0.0006	Y501:HE2	K353:CE
H-C	3.887	0.0002	Y501:HH	K353:CE
H-C	3.901	0.0001	Y501:HE2	F497:C
H-C	4.066	0.0005	Y501:HH	R498:CZ
H-C	4.086	0.0004	Y501:HB2	Q506:CB
H-C	4.191	0.0002	Y501:HD2	H505:CA
H-C	4.256	0.0003	Y501:HB2	Q506:CD
H-C	4.386	0.0004	Y501:HD1	Y41:CE2
H-C	4.400	0.0001	Y501:HB3	K353:C
H-C	4.412	0.0002	Y501:HB3	H505:CG
H-C	4.429	0.0001	Y501:HE1	R498:CG
H-C	4.434	0.0001	Y501:HA	D355:CA
H-C	4.437	0.0001	Y501:HA	D355:CG
H-C	4.482	0.0001	Y501:HE1	Y41:CB
H-H	3.644	0.0001	Y501:HD2	H505:HB2
H-H	3.737	0.0001	Y501:HB2	Q506:HE22
H-H	3.880	0.0002	Y501:HB2	H505:HB2
H-H	4.038	0.0001	Y501:HB3	Q506:HG2
H-H	4.054	0.0001	Y501:H	Q506:HG3
N...H	4.285	0.0001	Y501:N	D355:HB2
O...H	2.213	0.0016	Y501:HE2	S496:O
O...H	2.854	0.0016	Y501:H	R498:O
O...H	4.358	0.0002	Y501:HD2	S496:O
O...H	4.384	0.0001	Y501:HE2	H505:O
C-C	3.894	0.0003	Y41:CG	Y501:CE1
C-C	4.033	0.0013	Y41:CD2	Y501:CZ
C-C	4.082	0.0009	K353:CE	Y501:CZ
C-C	4.114	0.0007	K353:CE	Y501:CE2
C-C	4.123	0.0004	Y41:CG	Y501:CZ
C-C	4.133	0.0010	Y41:CE2	Y501:CZ
C-C	4.151	0.0002	F497:C	Y501:CE2
C-C	4.255	0.0014	S496:C	Y501:CE2

Table S17: N501 with their bonding for WT interface model.

N501				
C-C	4.318	0.0004	Q498:CA	N501:CG
C-O	3.697	0.0008	Q506:CD	N501:O
C-O	3.989	0.0002	Q498:CA	N501:OD1
H-C	3.914	0.0007	F497:HA	N501:CG
H-C	4.074	0.0004	Q506:HE22	N501:CA
H-C	4.272	0.0001	Y505:HB2	N501:CG
H-C	4.281	0.0001	Q506:HG2	N501:CA
H-C	4.402	0.0001	Y505:HB2	N501:C
H-C	4.429	0.0001	Q506:HA	N501:CG
H-C	4.458	0.0002	Q498:HB2	N501:CB
H-C	4.475	0.0001	Y505:HB2	N501:CA
H-H	3.210	0.0005	Q498:H	N501:HD22
H-H	3.359	0.0001	F497:HD1	N501:HD21
H-H	3.410	0.0001	K353:HD3	N501:HD22
N-C	4.064	0.0026	Q506:NE2	N501:C
N-C	4.220	0.0007	V503:N	N501:C
N-N	4.488	0.0005	P499:N	N501:N
O...H	1.869	0.0394	Q506:HE22	N501:O
O...H	2.429	0.0044	Q498:HB2	N501:OD1
O...H	3.553	0.0005	Q506:HE21	N501:O
O...H	3.828	0.0001	Q498:HB3	N501:OD1
O...H	4.252	0.0001	V503:HA	N501:O

Table S18: Y501 with their bonding for OV interface model.

Y501				
C-C	4.300	0.0013	Y41:CZ	Y501:CZ
C-C	4.363	0.0006	Y41:CE1	Y501:CZ
C-C	4.415	0.0001	K353:CB	Y501:CZ
C-C	4.435	0.0008	Y41:CE2	Y501:CD1
C-C	4.455	0.0001	R498:CG	Y501:CZ
C-C	4.464	0.0002	R498:CA	Y501:CZ
C-C	4.478	0.0009	K353:CG	Y501:CZ
C-C	4.481	0.0005	R498:CA	Y501:CE2
C-O	3.573	0.0019	K353:CE	Y501:OH
C-O	3.786	0.0009	Q506:CD	Y501:O
C-O	3.905	0.0014	R498:CZ	Y501:OH
C-O	4.359	0.0007	R498:CG	Y501:OH
C-O	4.389	0.0008	Y41:CZ	Y501:OH
C-O	4.426	0.0002	R498:CB	Y501:OH
C-O	4.471	0.0007	Y41:CE1	Y501:OH
H-C	3.688	0.0003	K353:HZ2	Y501:CZ
H-C	3.883	0.0021	K353:HZ1	Y501:CE1
H-C	3.994	0.0004	F497:HA	Y501:CZ
H-C	4.045	0.0001	H505:HB2	Y501:CG
H-C	4.066	0.0001	H505:HB2	Y501:CD2
H-C	4.116	0.0005	R498:HH11	Y501:CE1
H-C	4.189	0.0001	D355:HB2	Y501:C
H-C	4.205	0.0001	Q506:HG2	Y501:CA
H-C	4.259	0.0001	Q506:HG2	Y501:C
H-C	4.323	0.0003	K353:HZ1	Y501:CD2
H-C	4.365	0.0003	K353:HZ3	Y501:CZ
H-C	4.367	0.0001	H505:HB2	Y501:C
H-C	4.406	0.0002	R498:HB3	Y501:CE1
H-C	4.436	0.0002	H505:HB2	Y501:CA
H-C	4.461	0.0002	Y41:HD2	Y501:CZ
H-H	3.297	0.0005	R498:HD2	Y501:HH
H-H	3.660	0.0004	R498:HH12	Y501:HH
H-H	3.717	0.0001	F497:HD1	Y501:HE2
H-H	3.792	0.0001	K353:HZ3	Y501:HH
H-H	3.833	0.0001	R498:HB2	Y501:HH
H-H	3.932	0.0002	K353:HZ2	Y501:HH
H-H	3.934	0.0004	K353:HZ3	Y501:HE2
H-H	3.952	0.0001	K353:HE3	Y501:HH
H-H	4.061	0.0001	K353:HD3	Y501:HH
H-H	4.309	0.0001	D355:HB3	Y501:HA
N...H	4.100	0.0002	R498:NE	Y501:HH
N...H	4.355	0.0001	H505:N	Y501:HB3
N-C	3.589	0.0011	K353:NZ	Y501:CZ
N-C	3.996	0.0021	Q506:NE2	Y501:C
N-C	4.060	0.0010	V503:N	Y501:C
N-C	4.211	0.0014	R498:NH1	Y501:CZ
N-C	4.332	0.0002	R498:N	Y501:CE1
N-O	3.957	0.0008	R498:NE	Y501:OH
N-O	4.410	0.0001	R498:N	Y501:OH
O...H	1.782	0.0518	K353:HZ1	Y501:OH
O...H	1.864	0.0425	Q506:HE22	Y501:O
O...H	1.948	0.0086	R498:HH11	Y501:OH
O...H	3.434	0.0004	R498:HH12	Y501:OH
O...H	3.495	0.0003	Q506:HE21	Y501:O
O...H	3.575	0.0004	K353:HD3	Y501:OH
O...H	3.882	0.0001	V503:H	Y501:O
O...H	3.890	0.0002	K353:HE3	Y501:OH
O...H	3.985	0.0001	V503:HA	Y501:O
O...H	4.244	0.0001	Y41:HB2	Y501:OH

Table S19: Y505 with their bonding for WT interface model.

Y505				
C-C	3.707	0.0128	Y505:C	Q506:CB
C-C	3.841	0.0130	Y505:CA	Q506:CA
C-O	3.915	0.0008	Y505:CB	G504:O
C-O	4.019	0.0004	Y505:C	G504:O
H-C	3.787	0.0001	Y505:HB2	G504:C
H-C	4.047	0.0017	Y505:HA	G504:CA
H-C	4.140	0.0002	Y505:H	Q506:CA
H-H	3.087	0.0002	Y505:HA	Q506:H
N...H	3.123	0.0001	Y505:N	G504:HA3
N...H	3.208	0.0021	Y505:N	G504:HA2
N-C	1.360	0.4017	Y505:N	G504:C
N-C	4.176	0.0014	Y505:N	Q506:CA
O...H	2.398	0.0075	Y505:HA	G504:O
O...H	3.202	0.0083	Y505:H	G504:O
C-C	3.520	0.0046	G504:C	Y505:CB
C-C	3.587	0.0077	G504:C	Y505:C
C-C	3.853	0.0130	G504:CA	Y505:CA
C-O	3.459	0.0011	Q506:C	Y505:O
C-O	4.214	0.0006	Q506:CB	Y505:O
H-C	3.927	0.0016	Q506:H	Y505:CB
H-C	3.946	0.0008	Q506:HB2	Y505:C
H-C	4.084	0.0011	Q506:HA	Y505:CA
H-C	4.397	0.0001	Q506:HG2	Y505:C
H-H	3.286	0.0004	G504:HA3	Y505:H
H-H	3.442	0.0007	G504:HA2	Y505:H
N...H	3.800	0.0015	Q506:N	Y505:HB3
N...H	3.828	0.0004	Q506:N	Y505:HB2
N-C	1.346	0.4603	Q506:N	Y505:C
N-C	3.661	0.0034	Q506:N	Y505:CB
N-C	4.269	0.0007	G504:N	Y505:CA
O...H	2.444	0.0034	Q506:HA	Y505:O
O...H	3.171	0.0085	Q506:H	Y505:O
C-O	3.552	0.0014	Y505:CA	G502:O
C-O	4.187	0.0001	Y505:CG	K353:O
H-C	4.016	0.0010	Y505:H	G502:CA
H-C	4.169	0.0001	Y505:HE1	K353:C
H-C	4.261	0.0001	Y505:HD1	K353:CB
H-C	4.272	0.0001	Y505:HB2	N501:CG
H-C	4.402	0.0001	Y505:HB2	N501:C
H-C	4.475	0.0001	Y505:HB2	N501:CA
O...H	1.901	0.0335	Y505:H	G502:O
N-C	3.861	0.0006	Y505:N	G502:C
C-C	4.303	0.0002	K353:CB	Y505:CD1
C-C	4.348	0.0002	K353:C	Y505:CG
C-C	4.488	0.0001	K353:CA	Y505:CB
C-O	3.842	0.0007	N501:CG	Y505:O
H-C	3.891	0.0004	N501:HD22	Y505:CA
H-C	3.965	0.0002	K353:HB3	Y505:CD1
H-C	4.300	0.0002	K353:H	Y505:CE1
H-C	4.480	0.0001	P507:HD3	Y505:C
H-H	3.725	0.0002	N501:HD22	Y505:HB2
H-H	3.739	0.0001	N501:HD21	Y505:HB3
H-H	3.871	0.0001	K353:HB3	Y505:HD1
H-H	4.200	0.0001	N501:HB2	Y505:HB2
N...H	4.265	0.0001	N501:ND2	Y505:HB2
N-C	3.951	0.0008	N501:ND2	Y505:C
N-C	4.281	0.0004	P507:N	Y505:C
N-N	4.396	0.0005	V503:N	Y505:N
O...H	1.942	0.0359	N501:HD22	Y505:O
O...H	3.562	0.0005	N501:HD21	Y505:O
O...H	3.842	0.0001	P507:HD3	Y505:O

Table S20: H505 with their bonding for OV interface

H505				
C-C	3.719	0.0133	H505:C	Q506:CB
C-C	3.840	0.0133	H505:CA	Q506:CA
C-O	3.933	0.0004	H505:C	G504:O
C-O	4.020	0.0008	H505:CB	G504:O
H-C	3.861	0.0001	H505:HB2	G504:C
H-C	4.042	0.0016	H505:HA	G504:CA
H-C	4.217	0.0002	H505:H	Q506:CA
H-H	3.181	0.0005	H505:HA	Q506:H
H-H	3.833	0.0001	H505:HB2	Q506:H
N...H	3.233	0.0025	H505:N	G504:HA2
N...H	4.185	0.0001	H505:ND1	G504:HA3
N-C	1.359	0.4009	H505:N	G504:C
N-C	4.162	0.0014	H505:N	Q506:CA
O...H	2.390	0.0070	H505:HA	G504:O
O...H	3.198	0.0083	H505:H	G504:O
C-C	3.556	0.0063	G504:C	H505:C
C-C	3.568	0.0066	G504:C	H505:CB
C-C	3.853	0.0130	G504:CA	H505:CA
C-O	3.390	0.0009	Q506:C	H505:O
C-O	4.251	0.0006	Q506:CB	H505:O
H-C	3.833	0.0017	Q506:H	H505:CB
H-C	3.946	0.0008	Q506:HB2	H505:C
H-C	4.084	0.0010	Q506:HA	H505:CA
H-C	4.400	0.0001	Q506:HG2	H505:C
H-H	3.222	0.0004	G504:HA3	H505:H
H-H	3.484	0.0007	G504:HA2	H505:H
N...H	3.655	0.0005	Q506:N	H505:HB2
N...H	3.750	0.0016	Q506:N	H505:HB3
N-C	1.351	0.4335	Q506:N	H505:C
N-C	3.587	0.0027	Q506:N	H505:CB
N-C	4.288	0.0006	G504:N	H505:CA
O...H	2.496	0.0041	Q506:HA	H505:O
O...H	3.174	0.0086	Q506:H	H505:O
C-O	3.553	0.0005	H505:CD2	E37:OE1
C-O	3.653	0.0013	H505:CA	G502:O
C-O	3.661	0.0014	H505:CE1	E37:OE1
H-C	3.984	0.0007	H505:H	G502:CA
H-C	4.045	0.0001	H505:HB2	Y501:CG
H-C	4.066	0.0001	H505:HB2	Y501:CD2
H-C	4.100	0.0003	H505:HE2	K353:CB
H-C	4.367	0.0001	H505:HB2	Y501:C
H-C	4.404	0.0001	H505:HB3	K353:CG
H-C	4.436	0.0002	H505:HB2	Y501:CA
N...H	3.965	0.0001	H505:NE2	K353:HB3
N...H	4.355	0.0001	H505:N	Y501:HB3
N-C	3.918	0.0036	H505:NE2	E37:CD
N-C	4.071	0.0005	H505:NE2	K353:CB
N-C	4.417	0.0002	H505:NE2	K353:C
O...H	1.767	0.0460	H505:HE2	E37:OE1
O...H	1.981	0.0258	H505:H	G502:O
O...H	3.626	0.0004	H505:HD2	E37:OE1
O...H	3.842	0.0002	H505:HE1	E37:OE1
O...H	4.020	0.0010	H505:HE2	E37:OE2
C-C	4.420	0.0002	K353:C	H505:CD2
C-O	4.396	0.0002	Y501:CG	H505:O
C-O	4.478	0.0002	Y501:CE2	H505:O
H-C	3.845	0.0002	K353:HG2	H505:CG
H-C	4.072	0.0001	K353:HA	H505:CB
H-C	4.191	0.0002	Y501:HD2	H505:CA
H-C	4.202	0.0001	K353:HD3	H505:CG
H-C	4.412	0.0002	Y501:HB3	H505:CG
H-C	4.482	0.0001	K353:H	H505:CE1
H-H	3.644	0.0001	Y501:HD2	H505:HB2
H-H	3.792	0.0001	E37:HG3	H505:HE2
H-H	3.880	0.0002	Y501:HB2	H505:HB2
H-H	3.942	0.0001	K353:HA	H505:HB2
H-H	4.108	0.0001	K353:HG3	H505:HD2
H-H	4.230	0.0001	K353:HG2	H505:HB3
N-C	4.286	0.0003	P507:N	H505:C
N-N	4.348	0.0002	K353:N	H505:NE2
N-N	4.432	0.0004	V503:N	H505:N
O...H	3.933	0.0001	P507:HD3	H505:O
O...H	4.384	0.0001	Y501:HE2	H505:O

References:

1. VASP - Vienna Ab initio Simulation Package. Available online: https://www.vasp.at/ (acessed on 1 November, 2021).
2. Perdew, J.P.; K. Burke; M. Ernzerhof. Generalized gradient approximation made simple. *Physical review letters*, **1996**, *77*, 3865. https://doi.org/10.1103/PhysRevLett.77.3865.
3. Ching, W.-Y.; P. Rulis. Electronic Structure Methods for Complex Materials: The orthogonalized linear combination of atomic orbitals. **2012**, Oxford, UK: Oxford University Press.
4. Mulliken, R.S. Electronic population analysis on LCAO–MO molecular wave functions. I. *The Journal of Chemical Physics*, **1955**, *23*, 1833-1840. https://doi.org/10.1063/1.1740588.
5. Mulliken, R. Electronic population analysis on LCAO–MO molecular wave functions. II. Overlap populations, bond orders, and covalent bond energies. *The Journal of Chemical Physics*, **1955**, *23*, 1841-1846. https://doi.org/10.1063/1.1740589.
